# Mapping Protein Distribution in the Canine Photoreceptor Sensory Cilium and Calyceal Processes by Ultrastructure Expansion Microscopy

**DOI:** 10.1167/iovs.66.2.1

**Published:** 2025-02-03

**Authors:** Kei Takahashi, Raghavi Sudharsan, William A. Beltran

**Affiliations:** 1 Division of Experimental Retinal Therapies, Department of Clinical Sciences & Advanced Medicine, School of Veterinary Medicine, University of Pennsylvania, Philadelphia, PA, United States

**Keywords:** canine retina, connecting cilium, calyceal processes, photoreceptor sensory cilium, ultrastructure expansion microscopy

## Abstract

**Purpose:**

Photoreceptors are highly polarized sensory neurons, possessing a unique ciliary structure known as the photoreceptor sensory cilium (PSC). Vertebrates have two subtypes of photoreceptors: rods, which are responsible for night vision, and cones, which enable daylight vision and color perception. Despite the identification of functional and morphological differences between these subtypes, ultrastructural analysis of the PSC molecular architecture between rods and cones is still lacking. This study employed ultrastructure expansion microscopy (U-ExM) to characterize the PSC molecular architecture in canine retina.

**Methods:**

Canine neuroretinas (5-mm punches) were fixed in paraformaldehyde solution for either short or long durations. Additionally, 20-µm-thick cryosections from frozen archival retinal tissues fixed using the longer protocol were analyzed. A U-ExM protocol previously developed for mouse retina was adapted to these canine tissues with a battery of specific antibodies that label the various compartments of the PSC.

**Results:**

We demonstrated that U-ExM is applicable to both non-frozen and cryopreserved retinal tissues processed with standard paraformaldehyde fixation. Using this validated U-ExM protocol, we revealed the molecular localization of numerous ciliopathy-related proteins in canine photoreceptors. Furthermore, we identified significant architectural differences in the PSC, ciliary rootlet, and calyceal processes between canine rods and cones.

**Conclusions:**

U-ExM is a powerful tool for studying the PSC molecular architecture using frozen archival retinas that are processed following standard paraformaldehyde fixation and embedding protocols. The findings gained from this study pave the way for a better understanding of alterations in the molecular architecture of the PSC in canine models of retinal ciliopathies.

Photoreceptors are highly polarized sensory neurons located in the outermost layer of the neuroretina that play a crucial role in light detection and signal transduction. In vertebrate retinas, there are two subtypes of photoreceptors: rods, which are highly sensitive to light and essential for night vision, and cones, which provide high visual acuity, color perception, and daylight vision.^1^ These cells possess a unique sensory ciliary structure known as the photoreceptor sensory cilium (PSC). Within the PSC, photoreception and phototransduction occur in the outer segment (OS), a highly elaborated membrane structure supported by a ninefold microtubule axoneme that originates from the basal body (BB) in the inner segment (IS). The region connecting the OS and IS, termed the connecting cilium (CC), is characterized by Y-shaped linkers (Y-links) that anchor each microtubule axoneme to the cell membrane and function as a critical gateway for ciliary trafficking (Fig. 1).^2^^,^^3^ These fundamental features of the tubulin-based PSC architecture are thought to be shared between rods and cones, although detailed structural analyses for cone PSCs are still lacking.^4^

**Figure 1.**
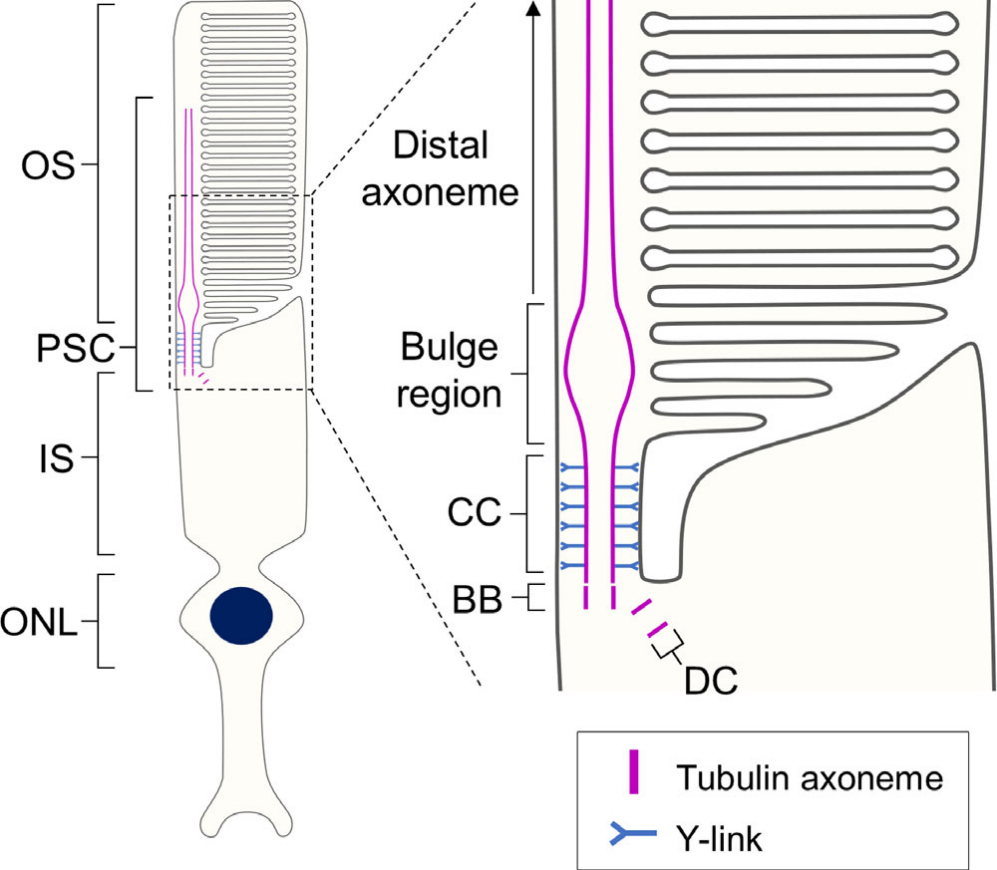
Schematic diagram of the rod photoreceptor sensory cilium. Overall view of the architecture of the rod photoreceptor (*left*) and detailed depiction of each compartment in the PSC. The PSC tubulin axoneme originates from the BB located in the IS and extends through the CC to reach the OS. Just beyond the CC, the axoneme expands at the bulge region and then tapers toward the distal part of the OS. BB, basal body; CC, connecting cilium; DC, daughter centriole; IS, inner segment; ONL, outer nuclear layer; OS, outer segment; PSC, photoreceptor sensory cilium.

Using cryo-electron tomography, the ultrastructure of rod PSCs has been thoroughly analyzed in the mouse retina, revealing that the CC structure of mouse rods is approximately 1.1 µm in length and 300 nm in diameter.^5^ Additionally, immunogold electron microscopy has been employed to investigate the precise localization of proteins within this small subcellular compartment, identifying the distribution of several ciliary proteins in mouse rod PSCs.^6^^–^^8^ However, despite the identification of pathogenic variants in over 50 genes associated with non-syndromic retinal ciliopathies,^9^ the specific roles and precise distribution of these proteins within the PSC have remained largely uncharacterized due to the limited resolution and detection capabilities of conventional fluorescence immunohistochemistry.

With recent advances in super-resolution imaging techniques, it has become feasible to analyze detailed protein localization and morphology within the PSC using fluorescence immunostaining.^10^^–^^13^ Notably, expansion microscopy (ExM), a sample preparation-based approach in which the specimen is expanded with a swellable hydrogel polymer and allows for ultrastructure observation without the need for specialized microscopy equipment, has become widely accessible to researchers.^14^ Numerous modified ExM protocols have been developed to optimize imaging for specific subcellular compartments and sample conditions,^15^ some of which have been successfully applied to investigate the ultrastructure of photoreceptors.^16^^–^^18^ Among these advanced methods, ultrastructure expansion microscopy (U-ExM) is particularly notable for its ability to preserve the native intracellular architecture of biological specimens while enabling nanometer-scale observation using standard primary and secondary antibodies used in immunohistochemistry.^19^ U-ExM has already made significant contributions to the molecular mapping of the mouse PSC and the detailed assessment of adeno-associated virus (AAV)-based gene augmentation therapy in mouse retinal ciliopathy models.^18^^,^^20^^,^^21^

Mice are frequently employed in research on PSC morphology and inherited retinal diseases (IRDs), including retinal ciliopathies, due to their ease of handling, low breeding costs, and suitability for gene editing. However, there are significant functional and structural differences between rodent and human photoreceptors, such as very low percentage of cones, the absence of a foveomacular region, and the lack of calyceal processes.^22^^–^^24^ To address these limitations and enhance the relevance of preclinical animal models, large animals such as dogs, cats, and pigs are proving to be valuable IRD models. Notably, canine models hold promise for advancing our understanding of the pathogenic mechanisms of IRDs, due to their fovea-like cone-rich structure^25^ and the identification of an increasing number of naturally occurring genetic mutations linked to human IRDs.^26^ Despite this potential, detailed morphological studies on canine photoreceptors remain limited. In this study, we utilized U-ExM to characterize the molecular architecture of the normal adult canine PSCs. We validated the U-ExM method for long-term cryopreserved canine retinal samples and provide a comprehensive analysis of the architectural features of the canine PSC. This includes differences in PSC structure between rods and cones, the distribution of retinal ciliopathy-related proteins, and the presence or absence of calyceal processes.

## Materials and Methods

### Animals

Retinal tissues from normal adult dogs were used for this study (Supplementary Table S1). All dogs were housed under identical conditions, including diet and ambient illumination with a 12-hour light/12-hour dark cycle, at the Retinal Disease Studies Facility (RDSF) at the University of Pennsylvania. The studies strictly adhered to the ARVO Statement for the Use of Animals in Ophthalmic and Vision Research and were approved by the Institutional Animal Care and Use Committee of the University of Pennsylvania.

### Tissue Processing for Long-Term Cryopreservation

The procedures for long-term cryopreservation of normal adult canine ocular tissues were performed as described in previous publications.^27^^,^^28^ The frozen tissues embedded in optimal cutting temperature (OCT) medium were stored at –80°C until cryosectioning was conducted. All cryopreserved samples used in this study had been stored for more than 12 years at –80°C (Supplementary Table S1).

### Immunohistochemistry on Non-Expanded Retinal Sections

The frozen OCT blocks were cryosectioned at a thickness 10 µm and stored at –20°C until further processing. The detailed methodology for immunostaining of non-expanded canine retinal cryosection has been described in a previous publication.^29^ Fluorescein-conjugated wheat germ agglutinin (WGA) was diluted 1:200 in Dulbecco's Phosphate-Buffered Saline (DPBS) and treated for 15 minutes after secondary antibody incubation. The reagents and antibodies used for conventional immunostaining are listed in Supplementary Tables S2, S3, and S4.

### Ultrastructure Expansion Microscopy on Canine Retina

Based on an ultrastructure expansion microscopy (U-ExM) protocol previously developed for mouse retina,^18^ we tested U-ExM on non-frozen and frozen archival canine retinal tissues. A schematic overview, photographs of each step, and reagents associated with the U-ExM protocol used in this study are summarized in Supplementary Figures S1 and S2 and in Supplementary Table S2.

#### U-ExM on Non-Frozen Canine Neuroretinas

After enucleation, the anterior part of eyeball, including the cornea, lens, and vitreous, was gently removed. The posterior eyecups were fixed in paraformaldehyde (PFA) solution for either short (4% PFA for 15 minutes) or long (4% PFA for 3 hours followed by 2% PFA for 24 hours) durations, and then 5-mm punches were taken from the eyecups using a disposable biopsy punch (Supplementary Fig. S2A). When it had been isolated from the retinal pigment epithelium (RPE)–choroid–sclera complex (Supplementary Figs. S2B, S2C), a 5-mm retinal punch was placed in a 14-mm microwell of a 35-mm Petri dish and then incubated overnight in 300 µL of 2% acrylamide (AA) + 1.4% formaldehyde (FA) solution at 37°C.

After removing the AA/FA solution, 45 µL of monomer solution (MS) composed of 25 µL of sodium acrylate (SA; stock solution at 38% [w/w] diluted with nuclease-free water), 12.5 µL of AA (40% solution), 2.5 µL of *N*,*N*′-methylenebisacrylamide (BIS; 2% solution), and 5 µL of 10× PBS was added to the microwell. After 90 minutes of incubation at room temperature, the MS was removed, and 90 µL of MS containing ammonium persulfate (APS) and tetramethylethylenediamine (TEMED) at a final concentration of 0.5% was dropped onto the tissue, followed by covering with a 12-mm circular cover glass (Supplementary Fig. S2D). Subsequently, the 35-mm Petri dish containing the tissue and MS solution was placed on ice for 45 minutes, followed by a 60-minute incubation at 37°C to allow gelation.

Next, 1 mL of denaturation buffer, comprised of 200-mM sodium dodecyl sulfate (SDS), 200-mM sodium chloride (NaCl), and 50-mM Tris base in deionized distilled water (ddH_2_O, pH 9), were added to the Petri dish for 15 minutes at room temperature with gentle agitation. The gel was carefully detached from the microwell using a spatula and then incubated in a 1.5-mL tube filled with denaturation buffer for 90 minutes at 95°C. Immediately after the denaturation step, the gel was transferred to a beaker filled with ddH_2_O, and the ddH_2_O was replaced twice, every 30 minutes, for the first round of expansion.

The following day, after measuring the diameter with a ruler (Supplementary Fig. S2E), the expanded gel containing retinal tissue was sliced with a razor blade to obtain approximately 1- to 2-mm-thick cross-sections. These sections were then placed in a well of a 24-well plate filled with DPBS containing calcium and magnesium (DPBS–Ca^2+^Mg^2+^) for the immunostaining steps.

#### U-ExM for Cryosections From Frozen Retinal Tissue

Cryosections, 20 µm thick, were obtained from the frozen posterior cup tissues that had been fixed with PFA using the longer protocol, cryoprotected in sucrose, and embedded in OCT media. The frozen sections on a slide glass were thawed at RT for 10 minutes (Supplementary Fig. S2F), and a square was drawn around the tissue section using a hydrophobic marker (ImmEdge Pen). After drying for 60 minutes at room temperature, 100 µL of DPBS–Ca^2+^Mg^2+^ was added into the hydrophobic square for 10 minutes to wash out the OCT compound.

After removal of DPBS, the sections were reacted with the AA/FA solution overnight, followed by penetration with MS for 90 minutes, as was done for non-frozen retinal punches. For each retinal section, 45 µL of MS containing APS and TEMED was dropped onto the tissue, and a 12-mm circular cover glass was placed on the solution (Supplementary Fig. S2G). After performing gelation, denaturation, and first round expansion as done for non-frozen retinal punches, the diameter of the gel was measured with a ruler (Supplementary Fig. S2H). The gel containing the retinal tissue was cut into approximately 1-cm squares using a razor blade and then placed in a well of a 24-well plate filled with DPBS–Ca^2+^Mg^2+^.

#### Immunohistochemistry on U-ExM Samples

After 10 minutes of incubation of the gels in DPBS, the solution in the well was replaced with 300 µL of blocking buffer containing the primary antibodies (Supplementary Table S3). The gels were incubated with the primary antibody solution overnight at room temperature with gentle shaking, followed by three washes with 1 mL of PBS with Tween 20 (PBST) for 5 minutes each. Alexa Fluor–conjugated secondary antibodies (Supplementary Table S4) were diluted in the blocking buffer at a concentration of 1:500 and incubated with the gel for 2 hours at room temperature, protected from light. Next, 100 µL of Hoechst 33342 solution, diluted 1:1000 in DPBS, was added to the wells and gently shaken for 30 minutes, followed by 3 washes with 1 mL of PBST. After the washes, each gel was transferred using a spatula to a well of a six-well plate filled with 10 mL ddH_2_O and incubated for 30 minutes to achieve the second round expansion. The ddH_2_O was replaced twice, every 30 minutes. The gels were then stored in ddH_2_O at 4°C, protected from light, until confocal imaging.

### Confocal Imaging

Prior to imaging, 14-mm microwells of 35-mm Petri dishes were filled with 0.1 mg/mL poly-d-lysine and kept at room temperature for 1 hour for coating to prevent drifting of the gels. After the poly-d-lysine solution was removed, the microwells were washed three times with ddH_2_O, dried, and then stored at 4°C. Image acquisition was performed on a STELLARIS 8 FALCON FLIM Microscope (Leica Microsystems, Wetzlar, Germany) using a 63× (1.20 NA) water objective. The lateral and axial views were obtained by orienting the expanded gel on the bottom of the microwell to face either the retinal cross-section or photoreceptor outer segment downward. The *z*-stacks for the lateral view were acquired with 0.2-µm *z* intervals, an *x*,*y* pixel size of 45.09 nm, and an imaging speed of 400 Hz. Representative images of the lateral view are shown as maximum projection images unless specified. Single images of the axial view were obtained with an *x*,*y* pixel size of 22.55 nm and an imaging speed of 100 Hz. The images were processed using Leica Application Suite X (LAS X) software for deconvolution with the LIGHTNING function, as well as for building merged and maximum projection images, and three-dimensional rendering.

### Quantification

#### Gel Diameter

The gel diameter after the first round expansion was measured using a ruler and then divided by the gel diameter before expansion (12 mm) to calculate the degree of gel expansion.

#### Expansion Factor

The expansion factor was calculated by dividing the width of the acetylated α-tubulin (AcTub)-labeled axoneme on the CC of U-ExM samples by the previously reported CC diameter of non-expanded mice photoreceptors (200.84 nm), quantified using cryo-electron tomography.^5^ A total of 40 photoreceptors in all U-ExM conditions were subjected to measurement of AcTub signal width using LAS X software manually. The width of the CC was consistent across all U-ExM conditions, and the expansion factor in this study was determined to be 3.83 by averaging the values of all U-ExM conditions. This expansion factor was used for every quantification.

#### Protein Signal Length, Width, and Distance From the Proximal End of the BB

Lengths, widths, and distance of protein signals were measured using the “Draw scalebar” function in LAS X software manually to fit the photoreceptor curvature. AcTub-labeled axoneme spread was measured at four different locations of the rod and cone photoreceptors corresponding to the CC or bulge region: +1000, +500, 0, and –500 nm. The distal end of the glutamylation signal located outside the tubulin axoneme was used to define the 0 location. Each measurement was subsequently corrected using the expansion factor.

### Statistical Analysis

Comparisons between two groups were performed using the nonparametric Mann–Whitney *U* test. Comparisons among more than three groups were conducted using the nonparametric Kruskal–Wallis test with Dunn's multiple-comparison test. Every measurement was performed on at least two individual eyes. Quantitative data are represented as scatter dot plots, with the center line and error bars in the graphs indicating the mean and standard deviation (±SD), respectively. The significance level is denoted as follows: ns, nonsignificant; *P* > 0.05, *P < 0.05, **P < 0.01, ***P < 0.001, and ****P < 0.0001. All statistical analyses were performed using Prism 10 (GraphPad, Boston, MA, USA). The data underlying the graphs shown in all the figures are included in the Supplementary Materials.

## Results

### U-ExM Protocol Is Effective on Non-Frozen and Cryopreserved Canine Retinal Tissues

To investigate whether U-ExM could be applied to canine retinal tissues, we tested a U-ExM protocol recently adapted for mouse retina^18^ on adult canine retinal tissues subjected to different fixation and storage conditions. The short-fixation condition (4% PFA for 15 minutes) was the same as that used for mouse retina in the previous U-ExM study, whereas a longer fixation protocol (4% PFA for 3 hours followed by 2% PFA for 24 hours) is routinely used by our lab for long-term cryopreservation of canine retinal tissues.

**Figure 2.**
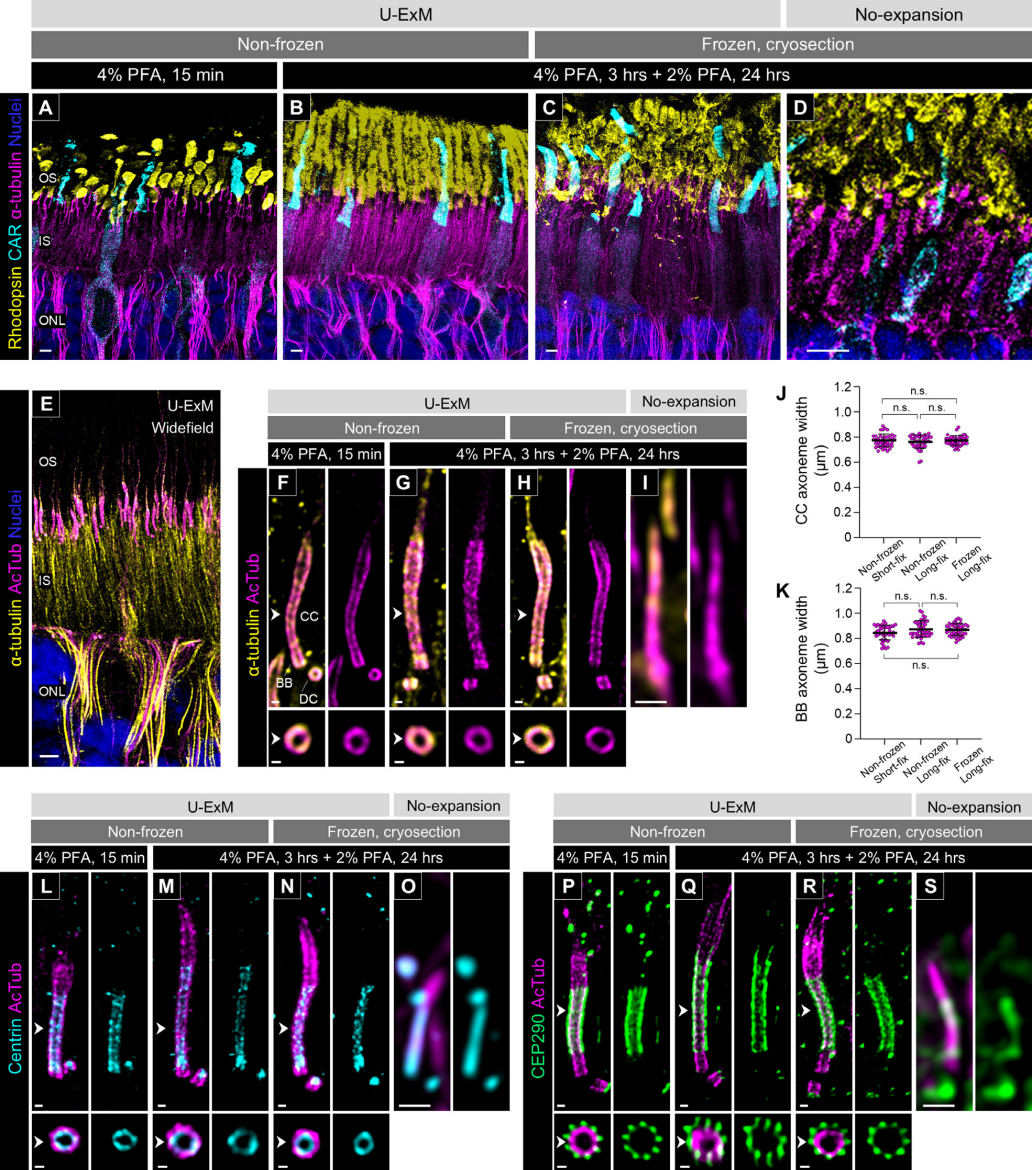
Comparison of tissue integrity and protein localization under different U-ExM conditions. (**A**–**D**) Low-magnification images of the outer retina stained for rhodopsin (*yellow*), CAR (*cyan*), and α-tubulin (*magenta*) in U-ExM samples subjected to different preservation/fixation protocols (non-frozen/short-fix, **A**; non-frozen/long-fix, **B**; frozen/long-fix, **C**) and non-expanded samples (**D**). Panel **D** is enlarged 3.8 times more than panels **A** to **C**. *Scale bars*: 5 µm, without correction for expansion factor. (**E**) Widefield view of a U-ExM sample stained for α-tubulin (*yellow*) and AcTub (*magenta*). *Scale bar*: 5 µm, without correction for expansion factor. (**F**–**I**) Details of α-tubulin (*yellow*) and AcTub (*magenta*) staining in the canine PSC under U-ExM (**F**–**H**) and non-expanded (**I**) conditions. Panel **I** is enlarged 3.8 times more than panels **F** to **H**. The lower panels show axial views at the CC for each U-ExM condition, indicated by *white arrowheads*. *Scale bars*: 500 nm (lateral view) and 200 nm (axial view), without correction for expansion factor. (**J**, **K**) Widths of AcTub-labeled CC (**J**) and BB proximal end (**K**) under different U-ExM conditions. Sample size: 40 photoreceptors. For each condition, four individual punches or cryosections from two to four eyes were stained. Non-significance (n.s., *P* > 0.05) was assessed by Kruskal–Wallis test with Dunn's multiple-comparison test. (**L**–**S**) Molecular localization of centrin (*cyan*, **L**–**O**) and CEP290 (*green*, **P**–**S**) in normal canine PSCs under U-ExM with different preservation/fixation protocols—non-frozen/short-fix (**L**, **P**), non-frozen/long-fix (**M**, **Q**), and frozen/long-fix (**N**, **R**) conditions—and under non-expanded (**O**, **S**) conditions. Panels **O** and **S** are enlarged 3.8 times more than panels **L** to **N** and **P** to **R**. *Scale bars*: 500 nm (lateral view) and 200 nm (axial view), without correction for expansion factor. INL, inner nuclear layer; PFA, paraformaldehyde.

First, we assessed the structural preservation of the photoreceptor OS after expansion under each sample condition. In non-frozen retinas fixed for the short duration, the OS were severely damaged and unevenly swollen (Fig. 2A). In contrast, the OS structures of both rods and cones were better preserved in the retinas fixed for the longer duration (Fig. 2B). Similarly, the OS of the cryosection-derived U-ExM samples exhibited relatively better preservation compared to the short-fixed non-frozen retina, although they showed mild damage (Fig. 2C). Because slight damage to the OS structure was observed even in the unexpanded cryosections (Fig. 2D), it is likely that the quality of the cryosectioning was reflected in the sample after expansion.

Next, to verify whether the expansion factor was affected by fixation duration and storage conditions in canine retinal tissues, we compared the structure of the tubulin axoneme of the PSC under each sample condition. Although non-acetylated tubulin has frequently been used as a PSC axoneme marker in previous U-ExM studies using mouse retina,^18^^,^^20^^,^^21^ this labeling also showed a distinct signal in the photoreceptor IS. In contrast, immunostaining with antibodies targeting AcTub, a widely used marker of primary cilia, labeled the PSC axoneme with high specificity (Fig. 2E). Furthermore, labeling of acetylated and non-acetylated tubulin at the BB and CC of the PSC showed identical localization in the magnified images of the U-ExM samples, although the non-acetylated tubulin signal was more prominent in the distal axoneme (Figs. 2F–H). This might suggest that the distal part of the PSC is less prone to acetylation than the BB and CC. We employed AcTub as a highly specific marker for the PSC axoneme in subsequent studies. The width of the AcTub-labeled axoneme on the CC and the proximal end of the BB after processing by U-ExM was comparable across all sample conditions (Figs. 2J, 2K). By dividing the CC axoneme width of U-ExM samples by 200.84 nm, which is the previously reported CC axoneme width of mouse photoreceptors,^5^ we determined an average expansion factor of 3.83 across all sample conditions. Similarly, the gel diameter immediately after the first round of expansion reached approximately 5 cm in all sample conditions (Supplementary Fig. S2I), averaging a 4.17 times larger dimension than the original gel diameter. These values are not notably different from the typical expansion factor (∼4) reported in previous U-ExM studies.^18^^,^^19^

We further investigated the localization patterns of representative CC-associated proteins to verify whether there were differences in the localization of these proteins among the different sample conditions. Centrin and centrosomal protein of 290 kDa (CEP290, or NPHP6), which have been well characterized in mouse photoreceptors as the CC inner scaffold protein and Y-link-associated protein, respectively,^18^ were examined. In U-ExM–processed canine photoreceptors, centrin localized specifically inside the tubulin axoneme of the BB and CC (Figs. 2L–N), whereas CEP290 was observed outside the CC axoneme (Figs. 2P–R), consistent with their previously reported localization patterns. Moreover, there were no significant differences in the localization of these proteins among the different sample conditions. In summary, our findings confirmed that U-ExM could be utilized for canine retinal tissue and that variations in PFA fixation and storage conditions do not affect the expansion factor. Considering the better preservation of OS structure, we used long-fixed non-frozen retinas and frozen archival retinal tissues for the subsequent studies described below.

### Differences in Tubulin-Based Molecular Architecture Between Rod and Cone PSCs

**Figure 3.**
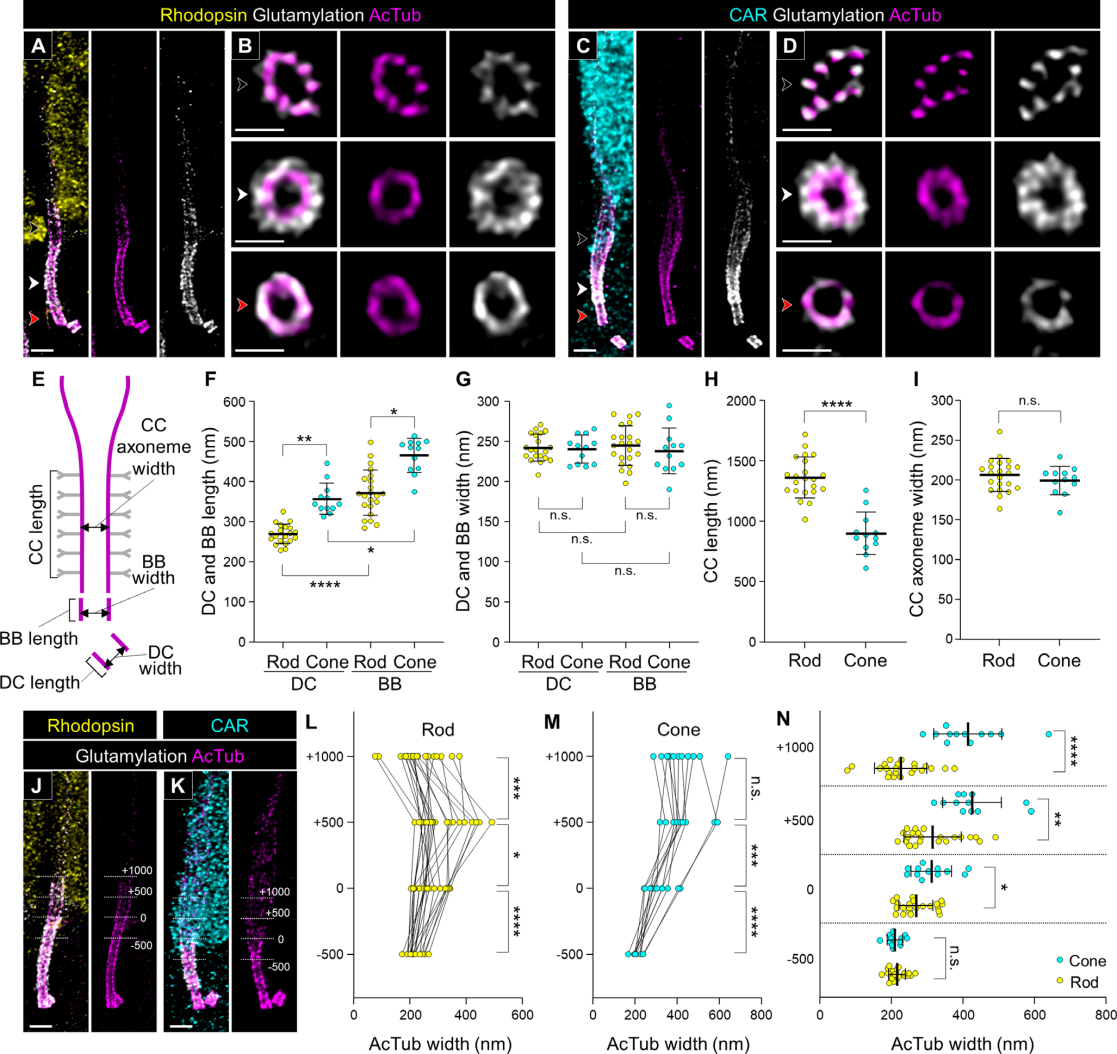
Tubulin-based molecular structure of rod and cone PSCs in normal adult canine photoreceptors. (**A**–**D**) Confocal images of expanded adult canine PSCs stained for AcTub (*magenta*) and glutamylated microtubules (*white*). Rod and cone photoreceptors were identified by labeling with rhodopsin (*yellow*, **A**) and CAR (*cyan*, **C**), respectively. *Black*, *red*, and *white arrowheads* on the bulge region, CC, and BB correspond to the respective axial views in panels **B** and **D**. *Scale bars*: 500 nm (lateral view) and 200 nm (axial view), after correction for expansion factor. (**E**) Schematic diagram showing the measurement of length and width in the CC, BB, and DC. The tubulin axoneme labeled with AcTub and the glutamylation signals on Y-links are represented in *magenta* and *gray*, respectively. (**F**–**I**) Comparison of length and width on the BB (**F**, **G**) and the CC (**H**, **I**) between rod and cone PSCs. (**J**, **K**) Rod (**J**) and cone (**K**) PSCs illustrating the measurements of AcTub-labeled axoneme width at four locations relative to the bulge region: +1000, +500, 0, and –500 nm. The distal end of the glutamylation signal located outside of the tubulin axoneme was used to define the 0 nm location. *Scale bars*: 500 nm, after correction for expansion factor. (**L**, **M**) Measurements of tubulin width in rod (**L**) and cone (**M**) PSCs at the four locations illustrated in panels **J** and **K**. (**N**) Comparison of tubulin width between rod and cone photoreceptors at the four locations shown in panels **J** and **K**. Sample size: 12 to 22 from ≥2 individual eyes. *P < 0.05, **P < 0.01, ***P < 0.001, and ****P < 0.0001, as assessed by Kruskal–Wallis test with Dunn's multiple-comparison test (**F**, **G**) or Mann–Whitney test (**H**, **I**, **L**–**N**).

We then analyzed the molecular architecture of the tubulin axoneme in rod and cone PSCs of normal adult canine retinas processed by U-ExM. Rod and cone photoreceptors were distinguished by labeling with rhodopsin and cone arrestin (CAR), respectively. The tubulin axoneme in both photoreceptor subtypes was visualized by labeling acetylation and glutamylation on the tubulin, which are well-known post-translational modifications in the centriole and primary cilium, using AcTub and GT335 antibodies. In both photoreceptor subtypes, glutamylation was detected on the tubulin axoneme throughout the PSC, similar to acetylation. The glutamylation signal was also found outside the tubulin axoneme only around the CC region (Figs. 3A–D). A recent study suggested that glutamylation signal shows localization comparable to CEP290 in mouse photoreceptor CC.^18^ Similarly, we identified that the region of the glutamylation signal on the tubulin axoneme matches the CEP290 signal on the CC in canine photoreceptors (Supplementary Fig. S3). Based on these findings, we assumed that the region with glutamylation outside the tubulin axoneme is equivalent to the CC and measured the length and width of each compartment of the PSC axoneme (Fig. 3E). As previously reported for other cell types,^30^^,^^31^ the length of the BB was significantly longer than that of the daughter centriole (DC) in each photoreceptor subtype. More interestingly, cone photoreceptors had notably longer DCs and BBs than rods (rod DC, 269.71 ± 24.00 µm; cone DC, 357.27 ± 37.26 µm; rod BB, 372.06 ± 55.22 µm; cone BB, 465.84 ± 40.58 µm) (Fig. 3F). In contrast, the length of the CC in cones was significantly shorter than that in rods (rod CC, 1362.81 ± 164.22 µm; cone CC, 900.35 ± 169.02 µm) (Fig. 3H). The widths of the AcTub-labeled tubulin axoneme in the CC, DC, and BB were identical among the photoreceptor subtypes (Figs. 3G, 3I).

Next, we focused on the differences between rod and cone tubulin structures in the outer region of the CC, referred to as the bulge region. Based on the measurement method used in previous studies on mouse photoreceptors,^18^^,^^21^ we measured the width of the tubulin axoneme at multiple positions along the CC in rod and cone photoreceptors: at the distal end of the CC (0), as well as 500 and 1000 nm above (+500, +1000) and 500 nm below (–500 nm) (Figs. 3J, 3K). In the rod PSC, the width of the tubulin axoneme significantly increased from the end of the CC to +500 nm, then decreased to +1000 nm (Fig. 3L). Conversely, in the cone PSC, the enhanced width of the tubulin axoneme from the CC endpoint to +500 nm was maintained even at +1000 nm (Fig. 3M). When comparing the axoneme width between rods and cones at each measurement point, the cone axoneme was significantly wider than the rod axoneme at all points except the middle part of the CC (–500 nm) (Fig. 3N). These results revealed that cone photoreceptors have longer and wider bulge region than rod photoreceptors.

### Molecular Mapping of the Bulge Region and Distal Axoneme in Canine Rod and Cone PSCs

**Figure 4.**
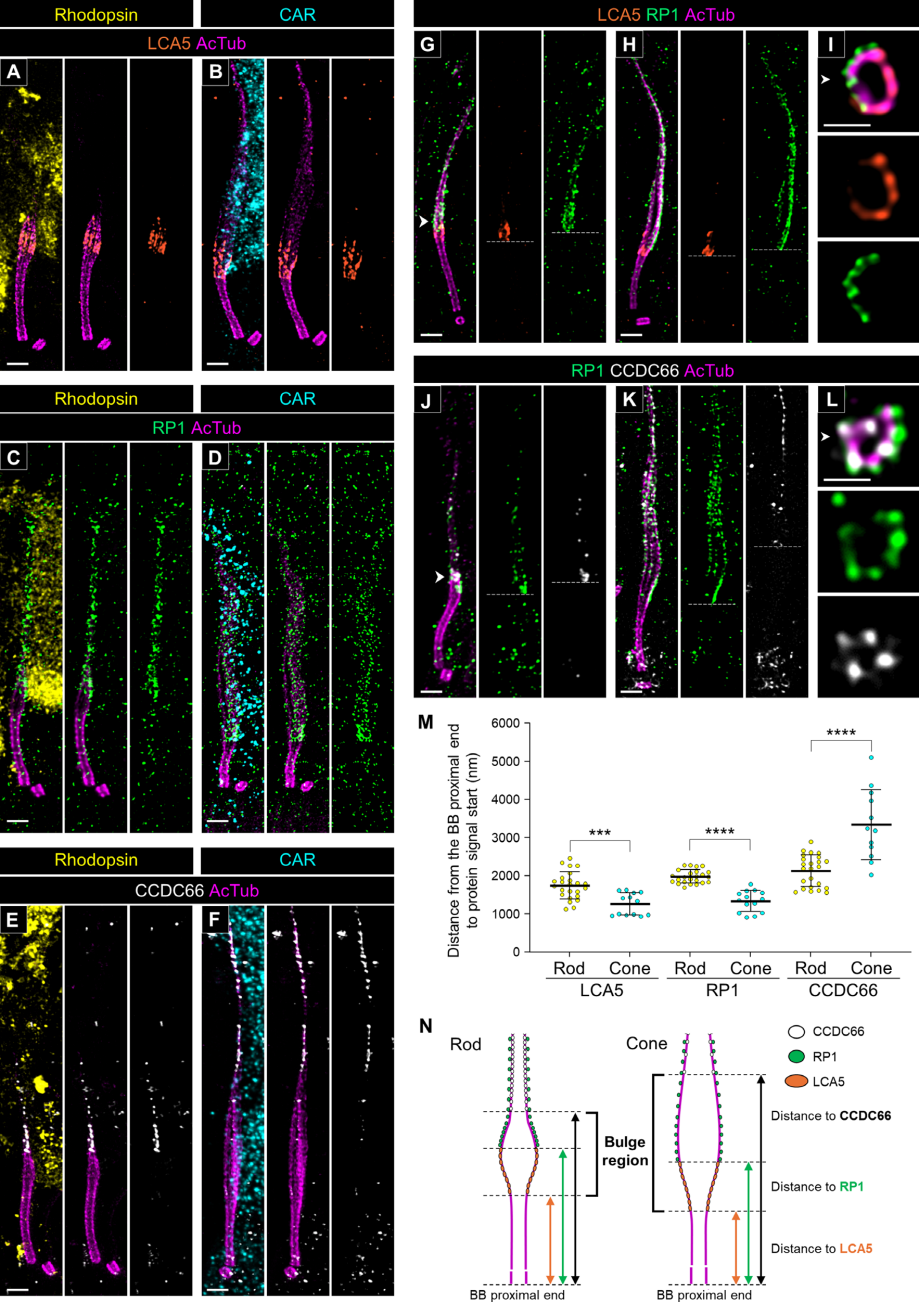
Molecular architecture of the bulge region and distal axoneme in normal canine rod and cone PSCs. (**A**–**F**) Lateral views of expanded adult canine PSCs stained for LCA5 (*orange*; **A**, **B**), RP1 (*green*; **C**, **D**), and CCDC66 (white; **E**, **F**) in rod (**A**, **C**, **E**) and cone (**B**, **D**, **F**) photoreceptors, respectively. CCDC66 was labeled using an Invitrogen CCDC66 polyclonal antibody (PA5-60642; Thermo Fisher Scientific, Waltham, MA, USA). *Scale bars*: 500 nm, after correction for expansion factor. (**G**–**L**) Co-immunolabeling with LCA5/RP1 (*orange*/*green*, **G**–**I**) and RP1/CCDC66 (*green*/*white*, **J**–**L**) in each photoreceptor subtype. The *right panels* (**I**, **L**) show axial views of the bulge region indicated by the *white arrowheads* in each staining. The *white dashed lines* represent the starting position of each protein signal on the tubulin axoneme. *Scale bars*: 500 nm (lateral view) and 200 nm (axial view), after correction for expansion factor. (**M**) Comparison of distance from the proximal end of the BB to the starting point of each protein signal between rod and cone PSCs. Sample size: 12 to 24 from ≥3 individual eyes. ***P < 0.001, ****P < 0.0001, as assessed by Mann–Whitney test. (**N**) Model illustrating the differences in molecular architecture of the distal part of the PSC, including the bulge region, between rod and cone photoreceptors.

To further characterize the differences in molecular architecture at the distal part of the tubulin axoneme between rods and cones in detail, we investigated the location of several proteins associated with the bulge region and distal axoneme in both rods and cones. Leber congenital amaurosis 5 protein (LCA5, or lebercilin) has been identified as a bulge region–specific protein in mouse photoreceptors.^18^^,^^21^ Consistent with the results from previous studies, LCA5 showed specific localization to the inner side of the bulge region in both rod and cone photoreceptors in the canine PSC (Figs. 4A, 4B). Additionally, retinitis pigmentosa 1 protein (RP1), known to localize from the bulge region to the distal axoneme in mouse photoreceptors,^21^ exhibited a comparable localization pattern in canine rod and cone photoreceptors (Figs. 4C, 4D). In this study, we identified coiled-coil domain containing 66 (CCDC66) as a novel protein that specifically localizes to the distal axoneme of both rods and cones (Figs. 4E, 4F). CCDC66 is a microtubule-binding protein, and dysfunction of its gene has been reported to cause retinal degeneration in both canine and mouse models.^32^^–^^34^ Although CCDC66 has been shown to localize to the BB and tubulin axoneme in cell lines,^35^ its distribution patterns in vertebrate retinal tissues have been inconsistent, with reports indicating localization in the OS and IS (Supplementary Table S5). In the present study, we tested three commercially available anti-CCDC66 antibodies, including those used in previous studies, on non-expanded canine retinas and found that their reactivity varied significantly (Supplementary Figs. S4A, S4C, S4E). However, in U-ExM samples, two of the three antibodies (PA5-60642 and sc-102418) produced specific signals on the PSC axoneme (Supplementary Figs. S4B, S4D, S4F). Although the sc-102418 antibody exhibited more extensive labeling than PA5-60642, both antibodies consistently showed a weak signal near the BB and a prominent signal at the distal axoneme.

To elucidate the positional relationship of these three proteins in the distal part of the PSC axoneme, we performed co-immunostaining of RP1 with LCA5 and CCDC66, respectively. In both rods and cones, LCA5 localized slightly more basally than RP1, and these two proteins did not co-localize (Figs. 4G, 4H). Additionally, axial view images showed that LCA5 localized on the tubulin axoneme, whereas RP1 localized outside the axoneme (Fig. 4I). CCDC66 appeared to overlap with RP1 in the distal axoneme in the lateral view (Figs. 4J, 4K). However, in the axial view, CCDC66 was localized on the tubulin axoneme, distinct from the localized area of RP1 (Fig. 4L). By measuring the distance from the proximal end of the BB to the starting point of each signal, we found that, in cones, LCA5 and RP1 localized more basally compared to rods, whereas CCDC66 localized more distally (Fig. 4M). Taken together, LCA5, RP1, and CCDC66 each exhibited unique localization patterns in the distal part of the PSC, and differences in localization of these proteins between rods and cones coincided with the architectural differences in the bulge region among these photoreceptor subtypes (Fig. 4N).

### Molecular Mapping of the CC in Canine Rod and Cone PSCs

**Figure 5.**
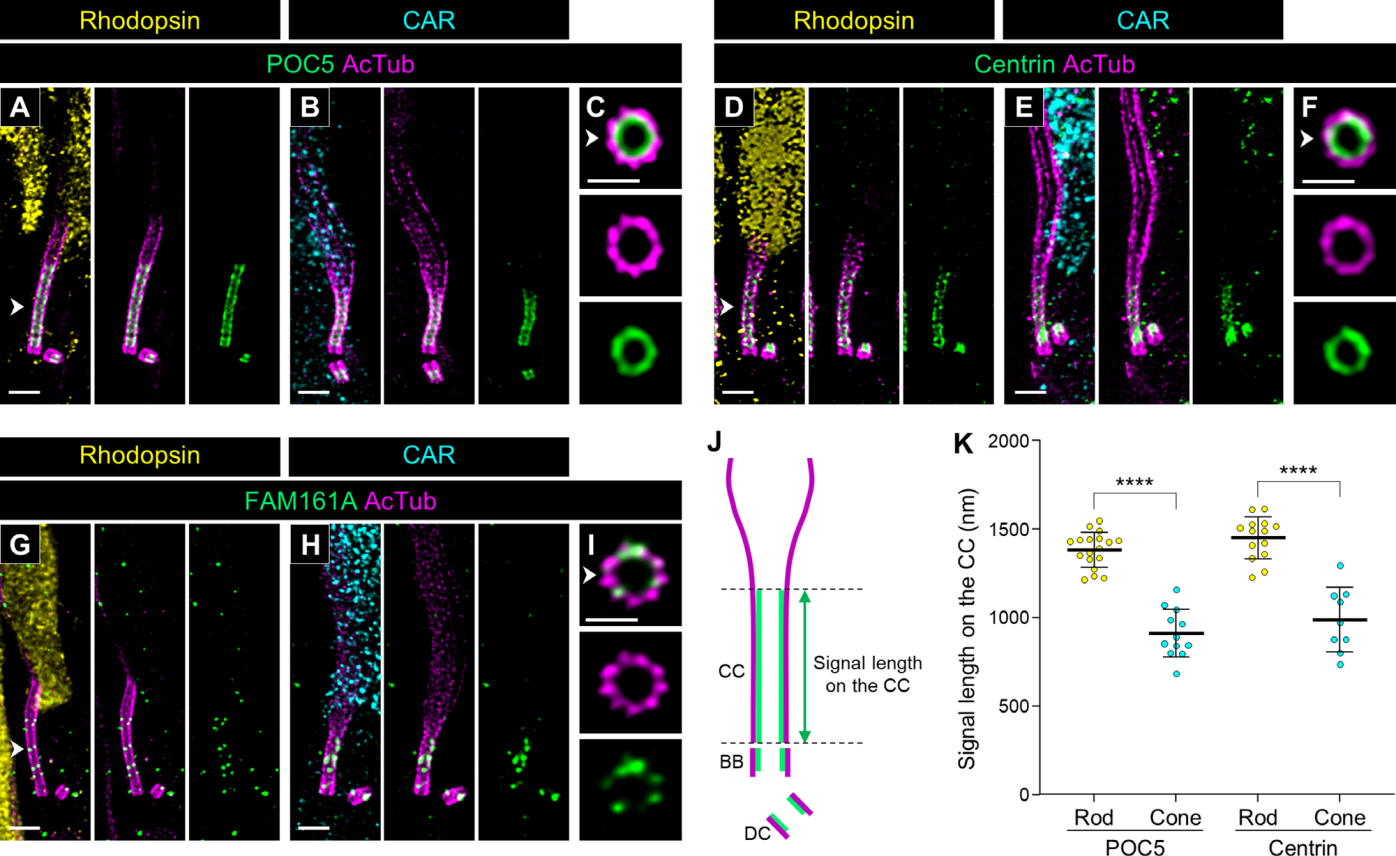
Location of inner scaffold proteins in normal canine rod and cone PSCs. (**A**–**I**) Confocal images of expanded adult canine PSCs stained for POC5 (*green*, **A**–**C**), centrin (*green*, **D**–**F**), and FAM161A (*green*; **G**, **H**). Rod and cone photoreceptors were identified by labeling with rhodopsin (*yellow*; **A**, **D**, G) and CAR (*cyan*; **B**, **E**, **H**), respectively. The *right panels* (**C**, **F**, **I**) show axial views of the CC for each protein, indicated by *white arrowheads*. *Scale bars*: 500 nm (lateral view) and 200 nm (axial view), after correction for expansion factor. (**J**) Schematic diagram illustrating the measurement of signal length in the CC. The tubulin axonemes labeled with AcTub and inner scaffold proteins are represented in *magenta* and *green*, respectively. (**K**) Comparison of POC5 and centrin signal lengths between rod and cone PSCs. Sample size: 9 to 18 from ≥2 individual eyes. ****P < 0.0001, as assessed by Mann–Whitney test.

Next, we compared the localization of CC-related proteins between rods and cones. Proteome of centriole 5 (POC5), centrin, and FAM161 centrosomal protein A (FAM161A) have been well characterized to localize inside the tubulin axoneme as inner scaffold proteins at the BB and CC in mouse photoreceptors.^18^ Consistent with the reports in mouse photoreceptors, these three inner scaffold proteins exhibited internal localization within the axoneme at the BB and CC in both canine photoreceptor subtypes (Figs. 5A–I). Because the endogenous FAM161A signal was too weak to quantify (Figs. 5G–I), similar to results in mouse photoreceptors,^18^^,^^20^ we measured the signal length in the CC for only POC5 and centrin (Fig. 5J). The signal lengths of these inner scaffold proteins at the CC were significantly shorter in cones than in rods, and these were consistent with the CC lengths measured in Figure 3 (rod POC5, 1383.09 ± 96.53 µm; cone POC5, 911.01 ± 129.72 µm; rod centrin, 1449.83 ± 115.57 µm; cone centrin, 989.27 ± 172.09 µm) (Fig. 5K).

**Figure 6.**
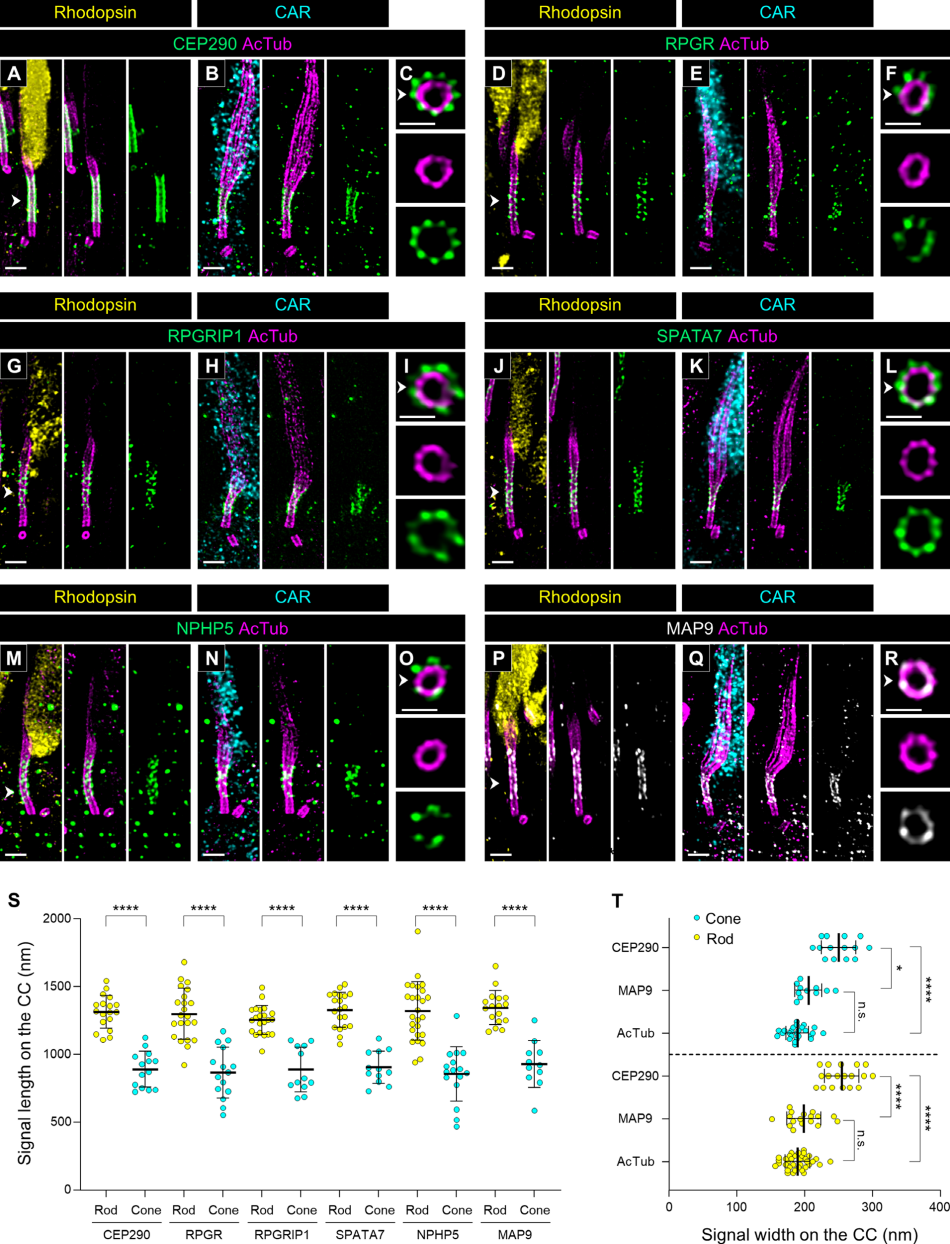
Molecular mapping of multiple connecting cilium-related proteins in normal canine rod and cone PSCs. (**A**–**R**) Expanded normal canine PSC stained for CEP290 (*green*, **A**–**C**), RPGR (*green*, **D**–**F**), RPGRIP1 (*green*, **G**–**I**), SPATA7 (*green*, **J**–**L**), NPHP5 (*green*, **M**–**O**), and MAP9 (*white*, **P**–**R**) in rod (**A**, **D**, **G**, **J**, **M**, **P**) and cone (**B**, **E**, **H**, **K**, **N**, **Q**) photoreceptors, respectively. Axial views of the CC for each protein, indicated by *white arrowheads*, are shown in panels **C**, **F**, **I**, **L**, **O**, and **R**. *Scale bars*: 500 nm (lateral view) and 200 nm (axial view), after correction for expansion factor. (**S**) Measurement of signal length of each protein on the CC in rod and cone PSCs. Sample size: 11 to 26 from ≥3 individual eyes. (**T**) Comparison of signal widths among CEP290, MAP9, and AcTub in rod and cone PSCs. Sample size: 11 to 35 from ≥3 individual eyes. *P < 0.05, ****P < 0.0001, as assessed by Mann–Whitney test (**S**) or Kruskal–Wallis test with Dunn's multiple-comparison test (**T**).

We then investigated the localization of several Y-link-associated proteins in canine photoreceptors. The Y-link is a hallmark element of the transition zone in the primary cilium, exhibiting a distinctive Y-shaped architecture that connects each microtubule doublet to the ciliary membrane.^3^ In mammalian photoreceptors, the CC is known as a region corresponding to the transition zone in the primary cilium, and CEP290, a typical Y-link component protein, has been confirmed to localize outside each of the nine tubulin axonemes in the CC of mouse photoreceptors.^18^ As shown in Figure 2 and Supplementary Figure S3, CEP290 localized at the CC in canine photoreceptors similar to its localization in mouse photoreceptors. Here, we also found that the signal length of CEP290 at the CC was significantly shorter in cones than in rods, consistent with the lengths of the CC shown in Figures 3 and 5 (rod CEP290, 1314.33 ± 118.71 µm; cone CEP290, 891.09 ± 126.21 µm) (Figs. 6A–C, 6S). Additionally, several other Y-link–associated proteins, including retinitis pigmentosa GTPase regulator (RPGR), RPGR interacting protein 1 (RPGRIP1, or LCA6), spermatogenesis associated 7 (SPATA7, or LCA3), and nephrocystin-5 (NPHP5, or IQCB1), were found to have signal lengths comparable to that of CEP290 at the CC in both rods and cones (Figs. 6D, 6E, 6G, 6H, 6J, 6K, 6M, 6N, 6S) and to localize outside the axoneme (Figs. 6F, 6I, 6L, 6O, 6R).

Finally, we found that microtubule-associated protein 9 (MAP9), a microtubule-binding protein, localizes specifically to the CC in photoreceptors (Figs. 6P, 6Q). A homozygous variant in the MAP9 gene has been reported to accelerate disease progression in a naturally occurring canine *RPGRIP1*-associated cone–rod dystrophy.^29^^,^^36^ The co-localization of MAP9 and RPGRIP1 in the CC of both photoreceptor subtypes suggests a direct interaction between these proteins. Additionally, axial view images and quantification of signal width at the CC revealed that MAP9 is located close to the tubulin axoneme, unlike the axial distribution of other Y-link–associated proteins (Figs. 6R, 6T). Mapping of these CC-specific proteins confirmed a significant difference in the length of the CC between canine rods and cones.

### Molecular Mapping of Ciliary Trafficking and Distal Appendage–Related Proteins on the Basal Bodies in Rod and Cone PSCs

**Figure 7.**
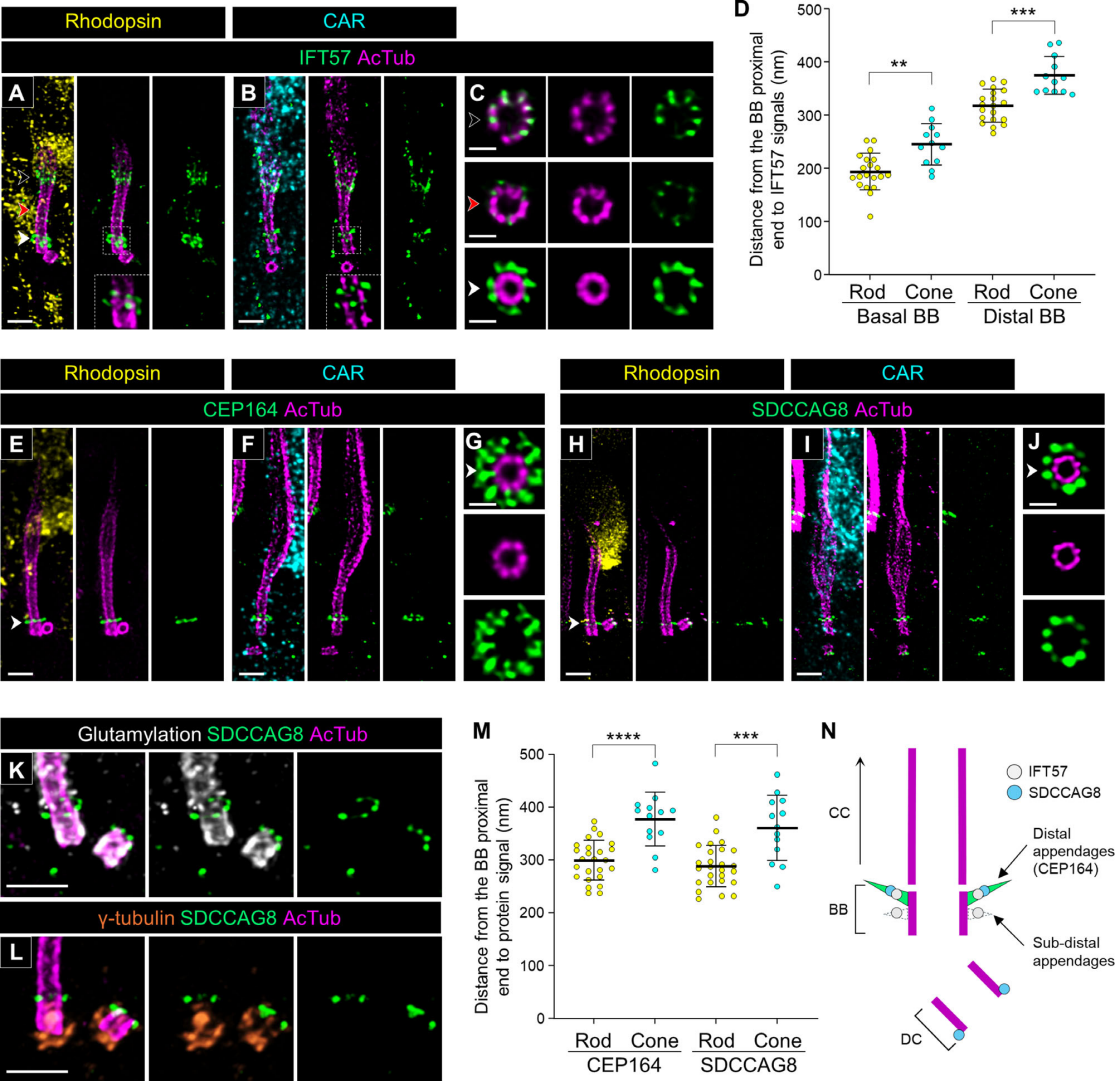
Localization of ciliary trafficking and distal appendage-related proteins in normal canine rod and cone PSCs. (**A**–**C**) Confocal U-ExM images of normal canine PSCs stained for AcTub (*magenta*) and IFT57 (*green*) in rod (**A**) and cone (**B**) photoreceptors, respectively. Insets, which are enlarged single *z*-scan images from panels **A** and **B**, highlight the detailed localization of IFT57 around the BB. *Black*, *red*, and *white arrowheads* on the bulge region, CC, and BB correspond to the axial views shown in panel **C**. *Scale bars*: 500 nm (lateral view) and 200 nm (axial view), after correction for expansion factor. (**D**) Comparison of distance from the proximal end of the BB to IFT57 signals around the basal part of PSCs between rod and cone PSCs. Sample size: 12 to 20 from ≥3 individual eyes. (**E**–**J**) Expanded normal canine photoreceptors labeled for distal appendage markers CEP164 (*green*, **E**–**G**) and SDCCAG8 (*green*, **H**–**J**) in rod (**E**, **H**) and cone (**F**, **I**) photoreceptors, respectively. Axial views of the basal part of PSCs for each protein, indicated by *white arrowheads*, are shown in panels **G** and **J**. *Scale bars*: 500 nm (lateral view) and 200 nm (axial view), after correction for expansion factor. (**K**, **L**) Co-immunolabeling with glutamylation/SDCCAG8 (*white*/*green*, **K**) and γ-tubulin/SDCCAG8 (*orange*/*green*, **L**). *Scale bars*: 500 nm, after correction for expansion factor. (**M**) Measurement of distance from the proximal end of the BB to the signal of each protein in rod and cone PSCs. Sample size: 13 to 26 from ≥3 individual eyes. **P < 0.01, ***P < 0.001, ****P < 0.0001, as assessed by Mann–Whitney test. (**N**) Model illustrating the identified location of IFT57 and SDCCAG8 around the distal appendages in normal canine photoreceptors.

We next mapped the localization of proteins associated with the BB, the compartment on the basal part of the tubulin-based PSC architecture. The intraflagellar transport (IFT) machinery is responsible for anterograde and retrograde trafficking of ciliary proteins and consists of two multisubunit complexes, IFT-A and IFT-B.^37^ In mouse photoreceptors, it was demonstrated using immunoelectron microscopy that several IFT particles have characteristic distribution around the BB and bulge region,^8^ and similar localization of these particles has recently been confirmed using U-ExM, as well^20^^,^^21^ We confirmed that, in both canine rods and cones, IFT57, a component of the IFT-B complex,^38^ has a similar distribution at the BB and bulge region as observed in mouse photoreceptors (Figs. 7A, 7B). Additionally, kinesin family member 3A (KIF3A), a component of kinesin-II, exhibited a distribution similar to that of the IFT particles in both photoreceptor subtypes, albeit with a high level of background signal (Supplementary Fig. S5). Axial view images showed that IFT57 distributes along each of the nine tubulin axonemes at both the BB and bulge region and is located closer to the axonemes at the bulge region than at the BB (Fig. 7C). We also found that the IFT57 signals at the BB were clearly divided into two layers (Figs. 7A, 7B). The distance from the proximal end of the BB to these signals was shorter in rods than in cones (Fig. 7D), consistent with the difference in BB length between the rods and cones presented in Figure 3.

Based on the characteristic distribution of IFT57 in the BB, we hypothesized that the IFT molecules localize around distal appendages, also known as transition fibers, in the BB, as has been suggested in previous publications.^39^^,^^40^ To verify this, we observed the distribution of centrosomal protein of 164 kDa (CEP164, or NPHP15), a representative distal appendage protein,^41^ in both rods and cones. As expected, CEP164 was localized in the distal part of the BB in both rods and cones (Figs. 7E, 7F), and the axial view exhibited a broader radial distribution (Fig. 7G), similar to that observed in recent studies using super-resolution microscopy techniques.^42^^,^^43^ The distance from the BB proximal end to CEP164 signals was shorter in rods than in cones, and these distances were comparable to these of the IFT57 signals at the distal BB (Figs. 7D, 7M).

Additionally, we found that Serologically defined colon cancer antigen 8 (SDCCAG8, or NPHP10), a retinal ciliopathy–associated protein,^44^^,^^45^ exhibits specific localization to the end of both BB and DC in rods and cones (Figs. 7H, 7I). The axial view images at the BB showed that SDCCAG8 has a ninefold symmetry distribution outside the axoneme, similar to IFT57 and CEP164 (Fig. 7J). Moreover, co-immunostaining of SDCCAG8 with glutamylation and γ-tubulin demonstrated that the SDCCAG8 signal on the ciliary axoneme is located between the BB and CC (Figs. 7K, 7L). The distance from the BB proximal end to SDCCAG8 was comparable to that of CEP164 in both rods and cones (Fig. 7M). Taken together, these results suggest that IFT57 and SDCCAG8 localize to distal appendages on the BB (Fig. 7N). Furthermore, based on the distance between distal and subdistal appendages suggested in previous study (∼100 nm), the location of the basal IFT57 signal in the BB is assumed to correspond to the location of the subdistal appendages.^46^

### Molecular Architecture of the Ciliary Rootlet in Canine Photoreceptors

**Figure 8.**
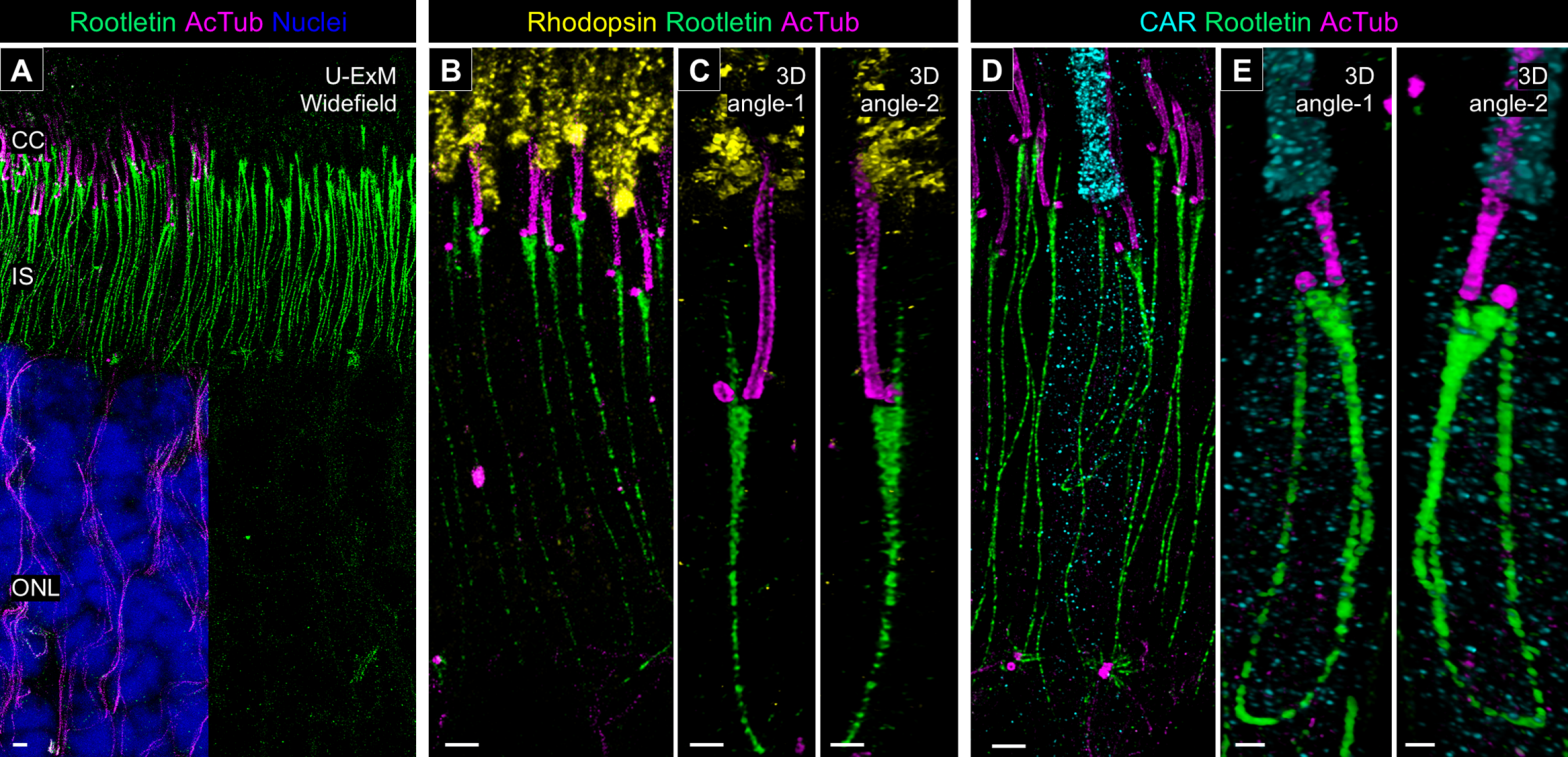
Structural characterization of the ciliary rootlet in normal canine rod and cone photoreceptors. (**A**) Widefield view of a U-ExM sample stained for rootletin (*green*) and AcTub (*magenta*). *Scale bar*: 1 µm, after correction for expansion factor. (**B**–**E**) High-magnification view of expanded normal canine PSCs stained for rootletin (*green*) and AcTub (*magenta*) in rod (**B**, **C**) and cone (**D**, **E**) photoreceptors, respectively. Three-dimensional renderings are shown in panels **C** and **E** from different angles. *Scale bars*: 1 µm (two-dimensional maximum projection) and 500 nm (three-dimensional view), after correction for expansion factor.

To address the peripheral structures of the PSC in canine photoreceptors, we mapped several proteins related to the ciliary rootlet. Rootletin (ciliary rootlet coiled-coil protein [CROCC]) is a 220-kDa coiled-coil protein identified as an architectural component of the rootlet in mouse photoreceptors^47^ and has been used as a marker of ciliary rootlet in several studies.^48^^,^^49^ Consistent with previous reports, in canine retinas processed with U-ExM, rootletin signals were observed as straight fibers in the photoreceptor IS, representing the architecture of the ciliary rootlets (Fig. 8A). In the magnified images, a rootlet stem extending straight to the BB and a characteristic branch-like structure extending to the middle of the CC were observed in the rod PSC (Figs. 8B, 8C, Supplementary Video S1). In contrast, cone photoreceptors exhibited a curvilinear rootlet architecture that was quite different from that of rods. In cone photoreceptors, the base of the rootlet did not contact the external limiting membrane, unlike in rods, and the ends of the rootlet appeared to be directed toward the BB and DC, respectively (Figs. 8D, 8E; Supplementary Video S2). These structural features in cones were consistent among cone photoreceptor subtypes, S-cones, and L-/M-cones (Supplementary Fig. S6).

### Molecular Architecture of the Calyceal Processes in Canine Photoreceptors

**Figure 9.**
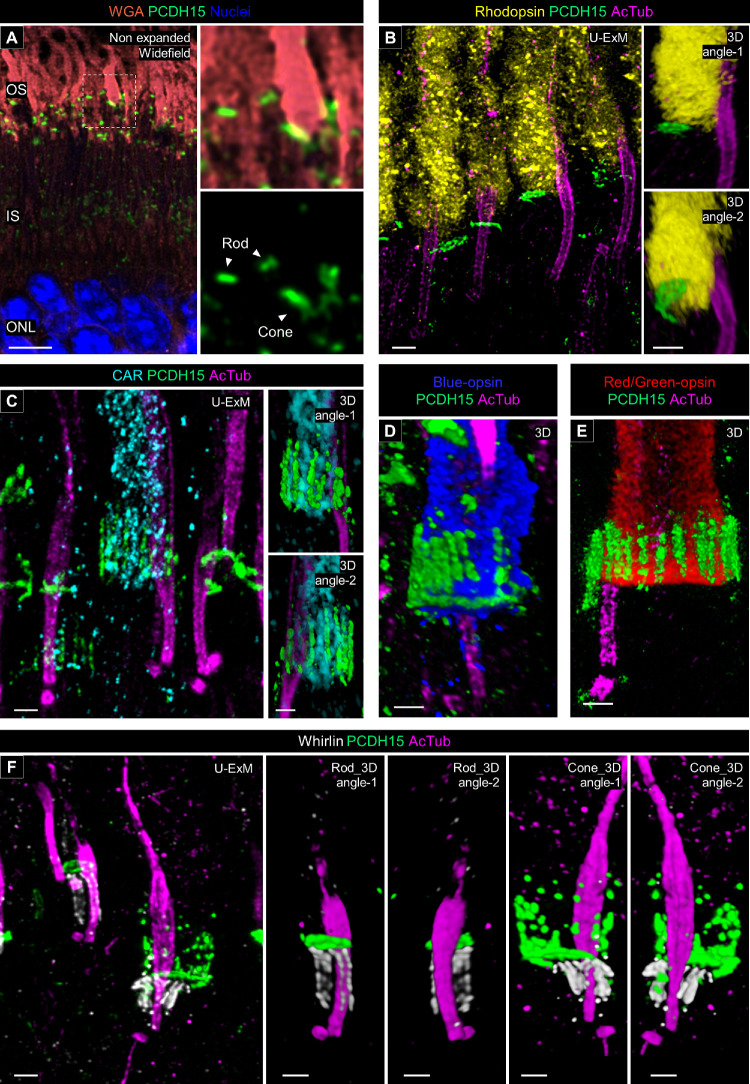
Molecular architecture of calyceal processes in normal canine photoreceptors. (**A**) Widefield view of a non-expanded retinal cryosection stained for WGA (*orange*) and PCDH15 (*green*). The *right panels* show details of PCDH15 location in rod and cone photoreceptors. *Scale bar*: 5 µm, without correction for expansion factor. (**B**–**E**) Confocal images of expanded adult canine photoreceptors stained for PCDH15 (*green*) and AcTub (*magenta*). Rod and cone photoreceptors were identified by labeling with rhodopsin (*yellow*, **B**), CAR (*cyan*, **C**), blue opsin (*blue*, **D**), and red and green opsin (*red*, **E**), respectively. The *right panels* in **B** and **C** and in **E** and **D** show three-dimensional renderings of one photoreceptor. *Scale bars*: 500 nm, after correction for expansion factor.

Finally, we investigated the localization of protocadherin-15 (PCDH15, or USH1F), an Usher 1 protein, to determine the presence or absence of calyceal processes in canine photoreceptors. Calyceal processes are microvilli-like structures characterized by the localization of Usher proteins in the apical region of the IS of human and nonhuman primate (NHP) photoreceptors and are absent in mouse photoreceptors.^24^ Recent research on NHP photoreceptors using expansion microscopy has described the structural organization of the calyceal processes based on the distribution of PCDH15.^17^ We followed this approach on our canine retinal tissues. First, we determined that PCDH15 is located between the OS and IS in both rods and cones in non-expanded retinal cryosections (Fig. 9A). In the canine rod photoreceptors processed by U-ExM, PCDH15 staining exhibited a characteristic C-shape, and its distribution was limited to the base of the OS (Fig. 9B, Supplementary Video S3). In contrast, PCDH15 formed a microvilli-like structure surrounding the OS near the OS/IS junction in the canine cones (Fig. 9C, Supplementary Video S4). The distribution pattern of PCDH15 was identical between S-cones and L-/M-cones (Figs. 9D, 9E). These differences in PCDH15 distribution between rods and cones were consistent with the previous observations in NHP photoreceptors.^17^ Additionally, we mapped Whirlin (USH2D), an Usher 2 protein, which localized to the basal side of PCDH15, along with the CC tubulin axoneme in both canine rods and cones. Whirlin exhibited a shorter distribution in cones compared to rods, correlating with the difference of CC length between these subtypes (Fig. 9F). We also attempted to localize another Usher 2 protein, very large G protein-coupled receptor 1 (VLGR1, or USH2C), in canine photoreceptors. However, identification of specific VLGR1 signals using a commercially available primary antibody was unsuccessful.

**Figure 10.**
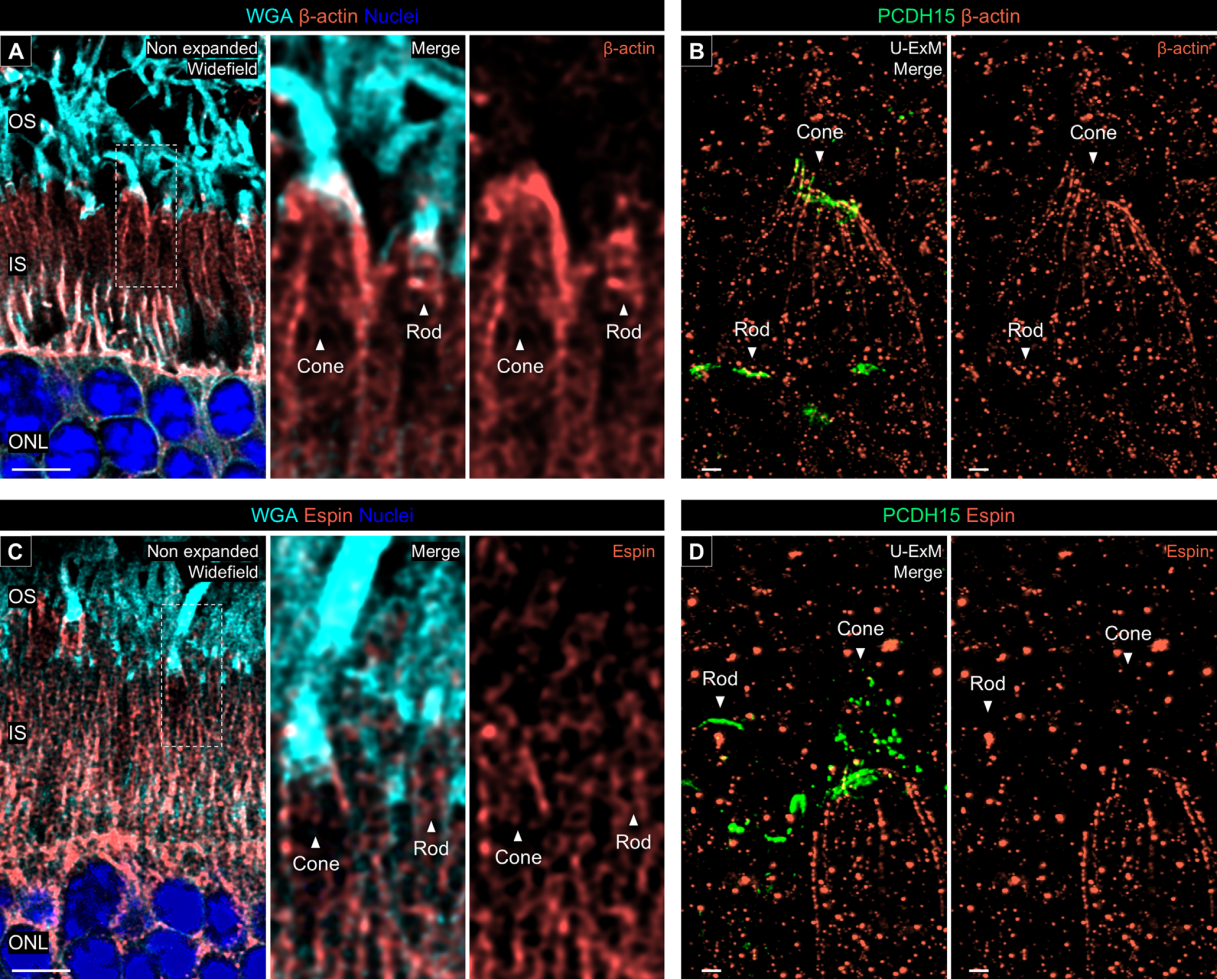
Actin filaments associated with calyceal processes. (**A**, **C**) Co-labeling with β-actin/WGA (*orange*/*cyan*, **A**) and espin/WGA (*orange*/*cyan*, **C**) in non-expanded canine retinal tissues. The *middle* and *right panels* show details of actin filaments location associated with calyceal processes in rod and cone photoreceptors. *Scale bar*: 5 µm, without correction for expansion factor. (**B**, **D**) Co-labeling with β-actin/PCDH15 (*orange*/*green*, **B**) and espin/PCDH15 (*orange*/*green*, **D**) in U-ExM samples of normal canine retina. *Scale bars*: 500 nm, after correction for expansion factor.

Finally, we localized actin filaments by immunolabeling of β-actin. In non-expanded canine retina, β-actin was distributed around the IS/OS interface in both cones and rods, although the signal intensity in rods was lower than in cones (Fig. 10A). However, in U-ExM samples, no actin filaments binding to PCDH15 were observed in canine rods, whereas canine cones displayed distinct actin filaments linking to the PCDH15-labeled microvilli-like architecture (Fig. 10B). These findings were further supported by espin labeling, which indicated that calyceal process-associated actin fibers in canine rods are more fragile than in cones, making them unable to maintain their architecture after expansion (Figs. 10C, 10D). Taken together, these results suggest that, in canine photoreceptors, whereas the localization of Usher proteins at the IS/OS junction is present in both rods and cones, the microvilli-like distribution of Usher 1 protein is found only in cone photoreceptors, similar to what is observed in NHP photoreceptors.

## Discussion

In the present study, we tested a U-ExM protocol to characterize the molecular architecture of the PSC using non-frozen and frozen archival canine retinal tissues. Utilizing archival samples, particularly from large animals such as dogs and NHPs, presents significant advantages in terms of cost efficiency and biological resource management. This approach not only is economical but also allows for the maximization of previously collected biological materials. However, as highlighted in the publication reporting the original U-ExM protocol, the type and duration of fixation can influence the degree of tissue expansion.^19^ Earlier studies applying U-ExM to the mouse retina typically used a short duration (15 minutes) of 4% PFA fixation.^18^^,^^21^ Here, we demonstrated that the duration of PFA fixation and long-term storage at –80°C did not significantly impact the degree of retinal tissue expansion, achieving approximately 4× isotropic expansion (Fig. 2). This observation aligns with the recent reports that applied U-ExM to PFA-fixed frozen and formalin-fixed paraffin-embedded mouse tissues.^20^^,^^31^ Notably, our results show that U-ExM enabled super-resolution imaging of archival samples that had been cryopreserved for over a decade, providing imaging quality comparable to that obtained from non-frozen samples. Although some physical damage to the OS membrane architecture was observed in the frozen section–derived U-ExM samples compared to the non-frozen samples, the tubulin axoneme structure remained unaffected across the sample processing conditions. Additionally, longer durations of PFA fixation (≥24 hours), which are routinely used for cryopreservation of ocular tissues, proved beneficial in preserving the structure of the photoreceptor OS in U-ExM. These findings underscore the robustness of U-ExM for use with archival samples, facilitating ultrastructural observation in large animal tissues where non-frozen samples are often challenging to procure.

In the molecular mapping of the PSC in normal adult canine retinal tissue, we discovered that the tubulin-based PSC architecture differs significantly between rods and cones (Fig. 3). Specifically, cones exhibited longer BBs and DCs, shorter CCs, and longer and wider bulge regions compared to rods. Additionally, the tubulin axonemes labeled by anti-AcTub were more prominent in the distal region of cones, which may suggest a more robust axoneme structure in cones than in rods. These structural differences were corroborated by the distinct localization patterns of proteins within each PSC compartment (Figs. 4^5^,^6^–7). Although detailed comparisons of PSC molecular architecture between rods and cones have been limited, our findings align with the known variations in the structural features of the OS between these photoreceptor subtypes.^50^ Previous studies have consistently reported that the CC length in mature mouse rods ranges from approximately 1100 to 1500 nm,^5^^,^^18^^,^^21^ which is consistent with the CC-specific protein signal lengths of approximately 1200 to 1400 nm observed in mature canine rods in this study. Similarly, the DC length in mature mouse rods is approximately 300 nm,^5^ closely matching the 270 nm measured in mature canine rods, indicating a conserved architecture of the CC and BB in rods across these species. However, comparisons of cone PSC architecture between canine and other vertebrates remain challenging due to a paucity of studies investigating the ultrastructure of cone PSCs to date. The significant architectural differences between rod and cone PSCs observed in this study underscore the necessity for further comprehensive investigations using retinal tissues from a variety of vertebrate species. These investigations are crucial to determine whether the architectural distinctions in the PSC between photoreceptor subtypes are unique to canines or represent a broader biological phenomenon.

An interesting question raised by the observed architectural variation between rod and cone PSCs is its involvement in the differential pathological progression among photoreceptor subtypes. A notable group of retinal ciliopathies, known as cone–rod dystrophy (CRD), is characterized by the progressive degeneration of cones preceding that of the rods. In canine models of naturally occurring retinal ciliopathy, homozygous variants in genes such as *NPHP4*, *NPHP5*, and *RPGRIP1* have been identified as causes of CRD,^51^^–^^53^ despite these genes being similarly expressed in both rods and cones. Previous studies have suggested that these CRD-associated proteins are localized to the CC in photoreceptors.^11^^,^^54^ Our current study confirms that NPHP5 and RPGRIP1 are indeed localized outside the tubulin axoneme at the CC in both photoreceptor subtypes (Fig. 6). Given their comparable distribution, these proteins are likely to have similar roles related to Y-links in both photoreceptor subtypes. Notably, the CC, where the tubulin axoneme of the PSC is anchored to the plasma membrane by Y-links, is significantly shorter in cones than in rods. This structural difference in the CC suggests that cones may be more susceptible to mutations in Y-link–associated genes compared to rods. The relationship between the heterogeneity of ciliopathy-associated phenotypes and the structural variations between rods and cones will be further investigated in future studies, which will involve integrating respective retinal ciliopathy models with U-ExM techniques to elucidate these differences.

Our study has also revealed significant differences in the architecture of the PSC rootlet between rods and cones (Fig. 8). Using U-ExM, we observed a linear shape of the rootlet in canine rods, which is consistent with the morphology of the rootlet in mouse rods, as previously described in transmission electron photomicrographs.^10^ A new finding in this study is the identification of a rootlet branch extending from the basal part of the PSC to the middle of the CC in rods. This branching structure has also been observed in recent transmission electron photomicrographs of canine photoreceptors, where it appears to be positioned on the opposite side of the PSC axoneme across the ciliary pocket,^55^ although its functional role remains unclear. Another interesting discovery is the unique elliptical shape of the cone rootlet. A recent study in NHP cones suggests that the cone rootlet has a markedly different shape from the commonly recognized form in the rod rootlet, although detailed morphological descriptions have been lacking.^17^ Here, we have demonstrated a curvilinear rootlet architecture in canine cones using U-ExM. Additionally, unlike the rod rootlet, which extends toward the external limiting membrane, the cone rootlet is entirely contained within the photoreceptor IS. Both rootlet structures likely play a critical role in providing stable intracellular support for the PSC and their associated OS structures. However, further studies are needed to elucidate the biological significance of these morphological differences between rod and cone photoreceptors.

During the mapping of various cilia-associated proteins using U-ExM, we determined that CCDC66 had a localization pattern that differed significantly from that reported in previous studies of the vertebrate retina. Earlier studies, based on immunolabeling of non-expanded retinal samples, suggested that CCDC66 localizes to the IS or OS.^32^^–^^34^ However, given the properties of CCDC66 as a microtubule-binding protein and previous findings from cell line studies,^35^ its localization to the OS or IS in photoreceptors seems unlikely. Using U-ExM, we found that two anti-CCDC66 antibodies (PA5-60642 and sc-102418) produced highly specific signals for the PSC axoneme, despite showing less specificity in non-expanded retinal tissue (Fig. 4, Supplementary Fig. S4). The PA5-46125 antibody, which did not detect signals in U-ExM, has a lower homology between its epitope and the corresponding canine CCDC66 protein region compared to PA5-60642 (74% vs. 85%, respectively), suggesting that this might affect its ability to accurately label the target protein. The improved specificity of the other anti-CCDC66 antibodies (PA5-60642 and sc-102418) for the PSC axoneme could be attributed to the enhanced accessibility of epitopes following the U-ExM procedure.

Finally, the application of U-ExM provided novel insights into the architecture of calyceal processes in canine photoreceptors (Figs. 9, 10). Our findings indicate that canine cones possess microvilli-like structures characterized by the distribution of PCDH15 around the basal part of the OS and the presence of associated actin filaments. In contrast, canine rods lack microvilli-like PCDH15 distribution, despite the localization of PCDH15 at the IS/OS junction. These differences in PCDH15 distribution between rods and cones are similar to those observed in photoreceptors of NHPs in a previous study.^17^ Interestingly, at the electron microscopy level, microvilli-like structures have been observed in NHP rods, though in smaller numbers compared to cones^24^; however, recent studies have shown an absence of microvilli-like PCDH15 distribution in these rods.^17^ This might suggest that the distribution of PCDH15 in rods does not align with the microvilli-like architecture considered as calyceal processes. To conclusively determine the presence or absence of calyceal processes in canine rods, further detailed morphological studies using electron microscopy are necessary. The localization of Usher proteins (PCDH15 and Whirlin), as well as actin filament–related proteins in canine photoreceptors, aligns with the patterns reported in NHP photoreceptors, suggesting that a canine IRD model may provide valuable insights into pathogenesis of Usher syndrome, for which effective pathological models are challenging to develop in rodents.

**Figure 11.**
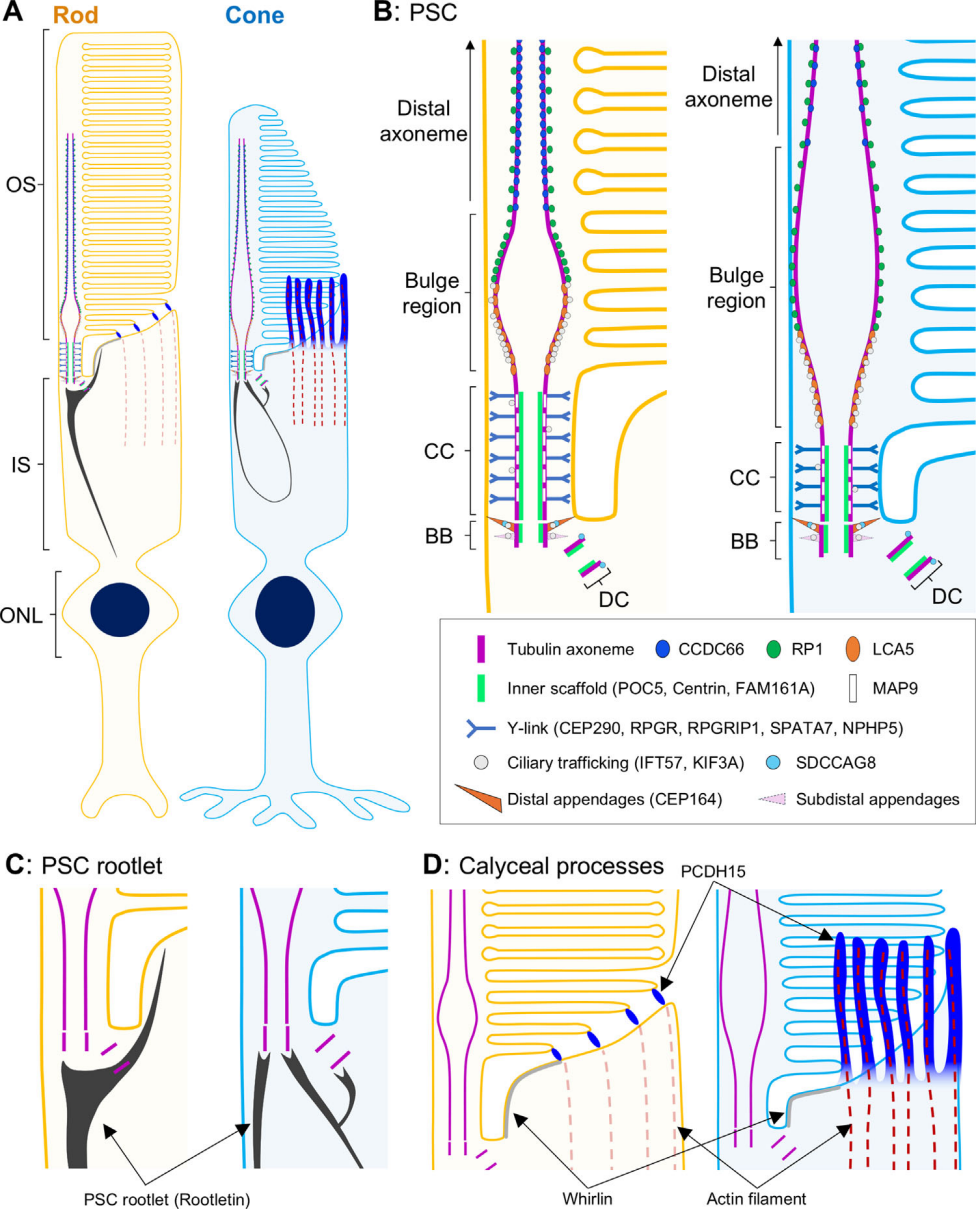
Schematic summary diagrams of the molecular architecture of PSC and calyceal processes and the distribution of retinal ciliopathy–associated proteins in normal canine rod and cone photoreceptors. (**A**) Overview of the architecture of normal canine rod (left) and cone photoreceptors. (**B**) Comparison of PSC molecular architecture between rod and cone photoreceptors. The cone PSC has a longer and wider bulge region, a longer BB, a longer DC, and a shorter CC than those of the rod PSC. (**C**) Structural differences of PSC rootlet between rod and cone photoreceptors. Rod photoreceptors have a straight rootlet extending toward the ONL, whereas the rootlet of cones forms a curvilinear shape. (**D**) Distribution of calyceal process–related proteins in rod and cone photoreceptors. In rod photoreceptors, the localization of PCDH15 is limited to the IS/OS junction, whereas in cone photoreceptors PCDH15 shows a microvilli-like distribution accompanied by actin filaments.

In conclusion, our study highlights the effectiveness of super-resolution imaging using U-ExM to characterize the molecular architecture of the canine PSC. By employing U-ExM, we have achieved detailed molecular mapping of numerous retinal ciliopathy–related proteins and have identified significant structural differences in the PSC between rods and cones in canine photoreceptors (Fig. 11). These insights gained from this study pave the way for a better understanding of the alterations in PSC molecular architecture and the efficacy of AAV-based gene augmentation therapies in established canine models of retinal ciliopathy, such as those with mutations in *RPGR*,^56^ *NPHP5*,^51^ *RPGRIP1*,^52^ or *CCDC66*,^34^ using U-ExM.

## Supplementary Material

Supplement 1

Supplement 2

Supplement 3

Supplement 4

Supplement 5

Supplement 6

Supplement 7
