# Supplementary material for: Mapping Protein Distribution in the Canine Photoreceptor Sensory Cilium and Calyceal Processes by Ultrastructure Expansion Microscopy
Source: Invest Ophthalmol Vis Sci. 2025 Feb 3;66(2):1. doi: 10.1167/iovs.66.2.1 (PMC11798334; doi:10.1167/iovs.66.2.1)
Supplement: Supplement 1 [file iovs-66-2-1_s001.pdf]

Supplementary Figure 1

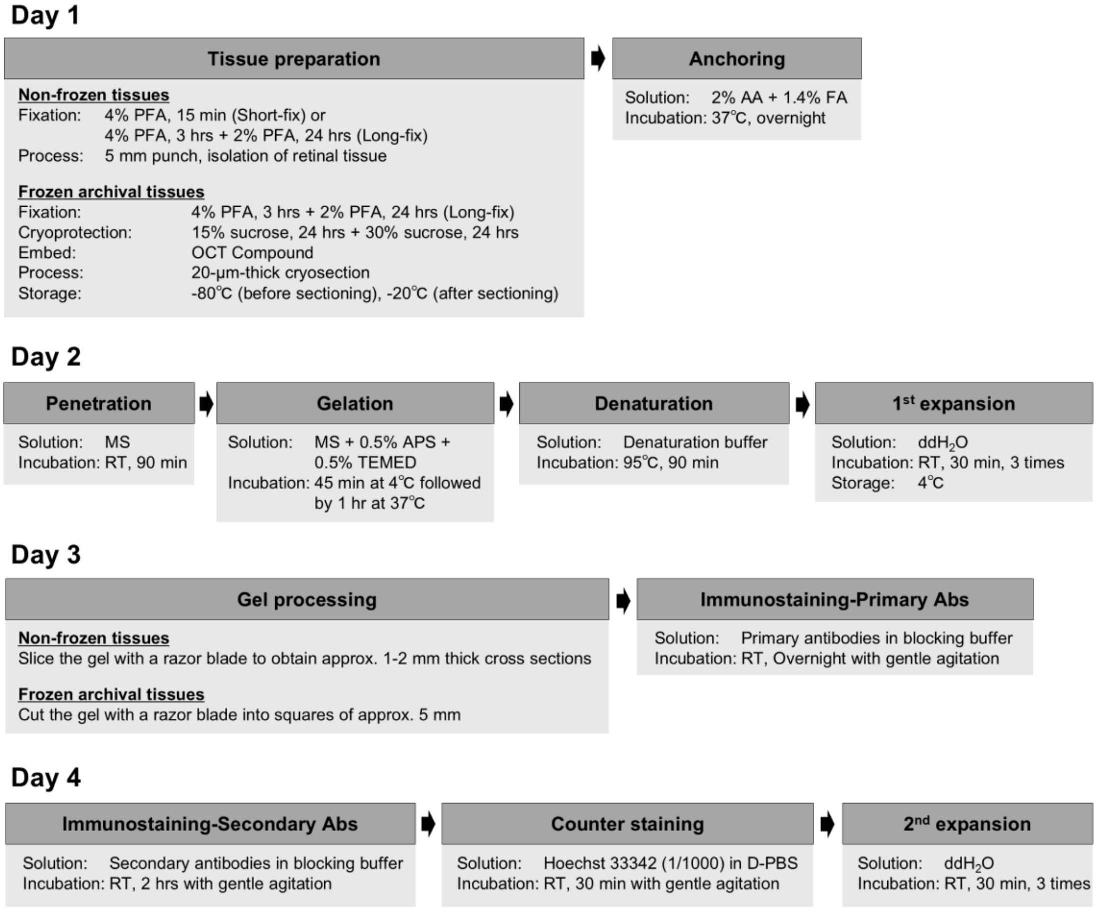

Supplementary Figure 1. Overview of the modified U-ExM protocol for non-frozen and frozen-archival canine retinal tissues

AA, acrylamide; ASP, ammonium persulfate; ddH_2O, distilled deionized water; FA, formaldehyde; MS, monomer solution; OCT, optimal cutting temperature; PFA, paraformaldehyde; RT, room temperature; TEMED, tetramethylethylenediamine.

Supplementary Figure 2

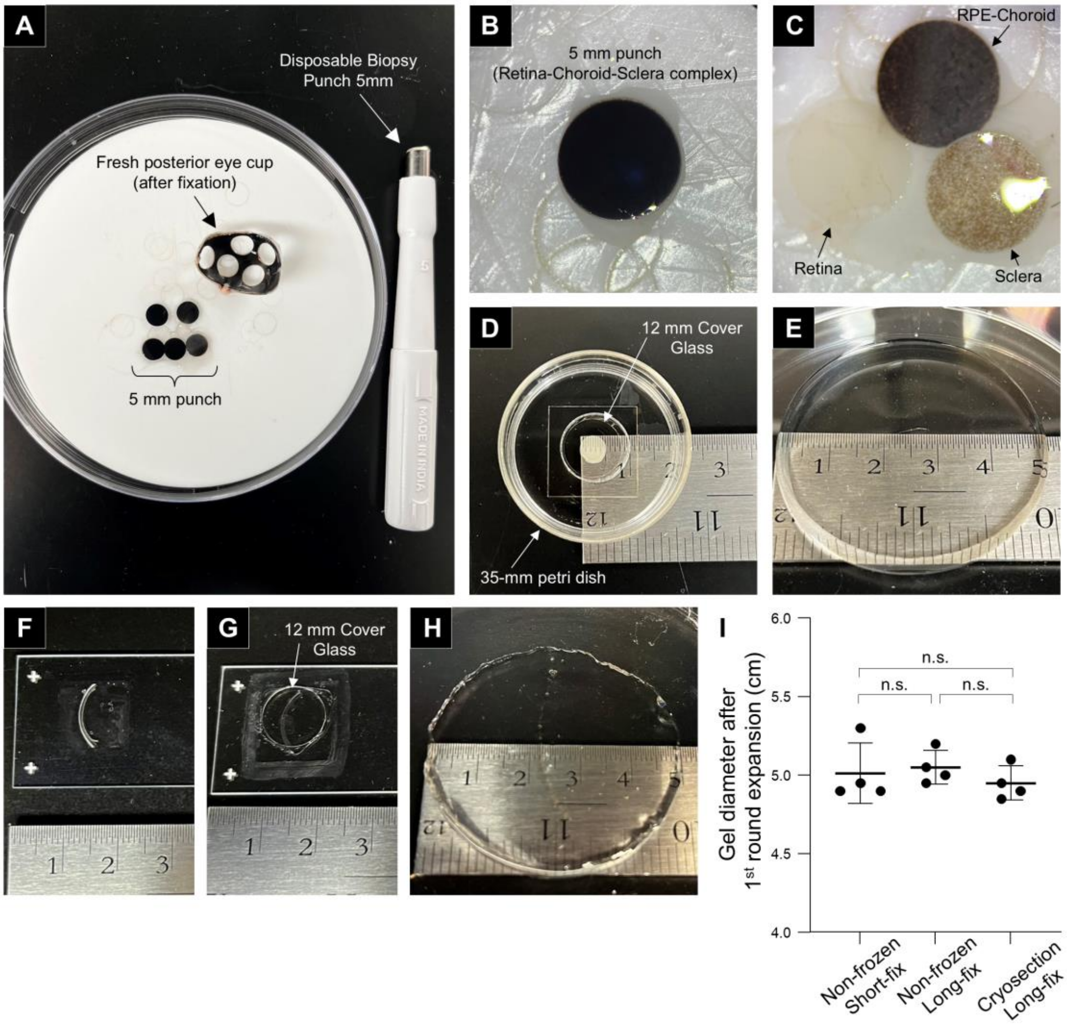

Supplementary Figure 2. Tissue processing and gel expansion in U-ExM with canine retinal tissues

(A) Collection of 5 mm tissue punches from PFA fixed- non-frozen canine posterior eyecup using a disposable biopsy punch. **(B, C)** Isolation of retinal tissue from the 5 mm tissue complex (B, before; C, after isolation). **(D)** Gelation of the 5 mm retinal punch using a 12 mm circular cover glass. **(E)** First-round expansion of the gel including the non-frozen retinal punch. **(F)** Twenty-μm-thick canine retinal

cryosection. **(G)** Gelation of the canine retinal cryosection using a 12 mm circular cover glass. **(H)** First-round expansion of the gel including the retinal section. **(I)** Comparison of gel diameter after the first-round expansion between different fixation conditions. Sample size: n = 4 individual punches or cryosections from 2-4 eyes per fixation/storage condition. Non-significant (n.s., $P > 0.05$) by Kruskal-Wallis test with Dunn's multiple-comparison test.

Supplementary Figure 3

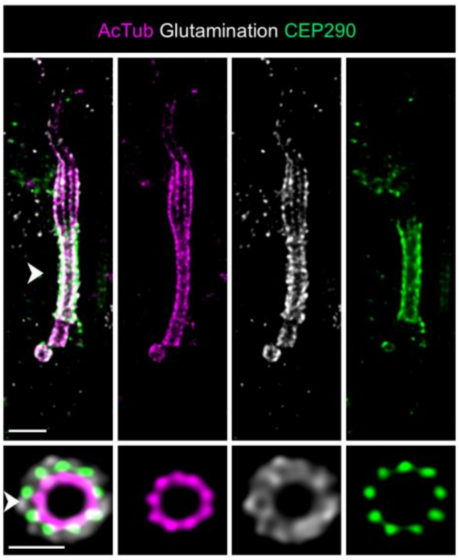

Supplementary Figure 3. Co-immunolabeling of CEP290 and glutamination on the connecting cilium of normal canine photoreceptors

Confocal images of expanded adult canine PSC stained for AcTub (magenta), glutamination (white), and CEP290 (green). The lower panels show axial views of the CC, indicated by white arrowheads. Scale bars: lateral view = 500 nm; axial view = 200 nm, after correction for expansion factor. Abbreviations: CC, connecting cilium.

Supplementary Figure 4

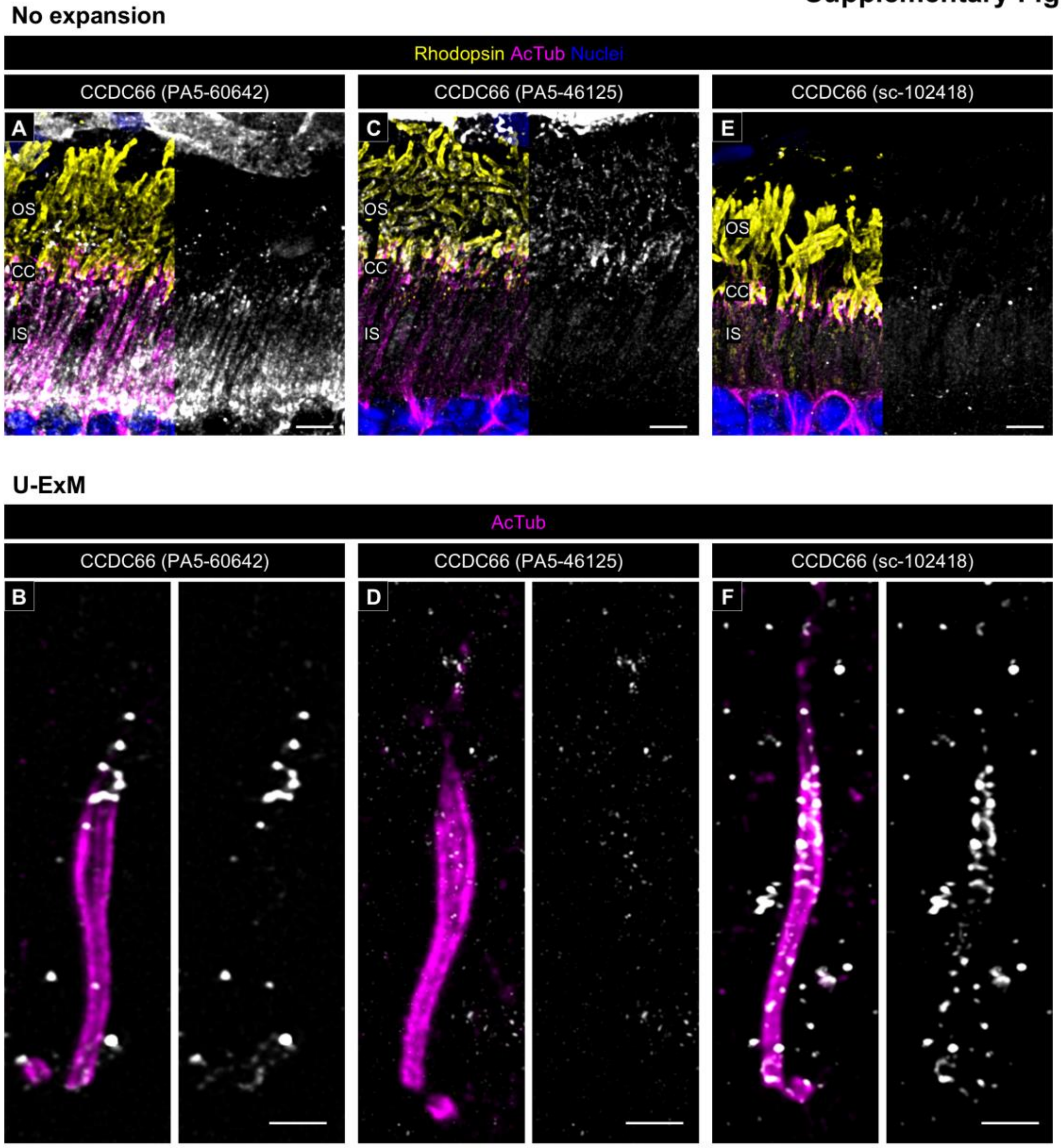

Supplementary Figure 4. Evaluation of the reactivity of multiple anti-CCDC66 antibodies in canine retinal tissues

(A, C, E) Confocal images of non-expanded retinal cryosections stained for rhodopsin (yellow), CCDC66 (white), and AcTub (magenta). Panels (A), (C), and (E) show the results of IHC using three different anti-human CCDC66 antibodies: PA5-60642, PA5-46125, and sc-102418, respectively. Scale bars: 5 μm, without correction for expansion factor. **(B, D, F)** Expanded normal canine PSC

labeled with the PA5-60642 antibody (white, B), PA5-46125 antibody (white, D), and sc-102418 antibody (white, F). The PSC axoneme is visualized by AcTub labeling (magenta). Scale bars: 500 nm, after correction for expansion factor. Abbreviations: CC, connecting cilium; IS, inner segment; OS, outer segment, PSC, photoreceptor sensory cilium.

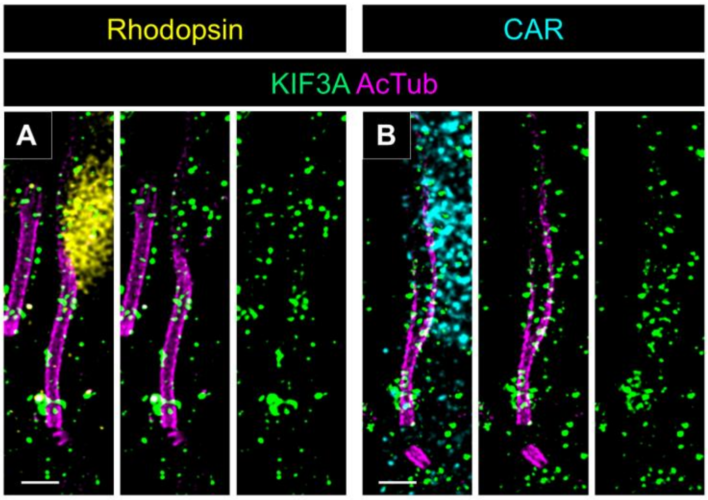

Supplementary Figure 5. Localization of KIF3A in normal canine rod and cone PSC

(A, B) Confocal U-ExM images of normal canine PSC stained for AcTub (magenta) and KIF3A (green) in rod (A) and cone (B) photoreceptors, respectively. KIF3A signal shows a similar localization pattern to IFT57 around the BB and bulge region. Abbreviations: BB, basal body; PSC, photoreceptor sensory cilium.

Supplementary Figure 6

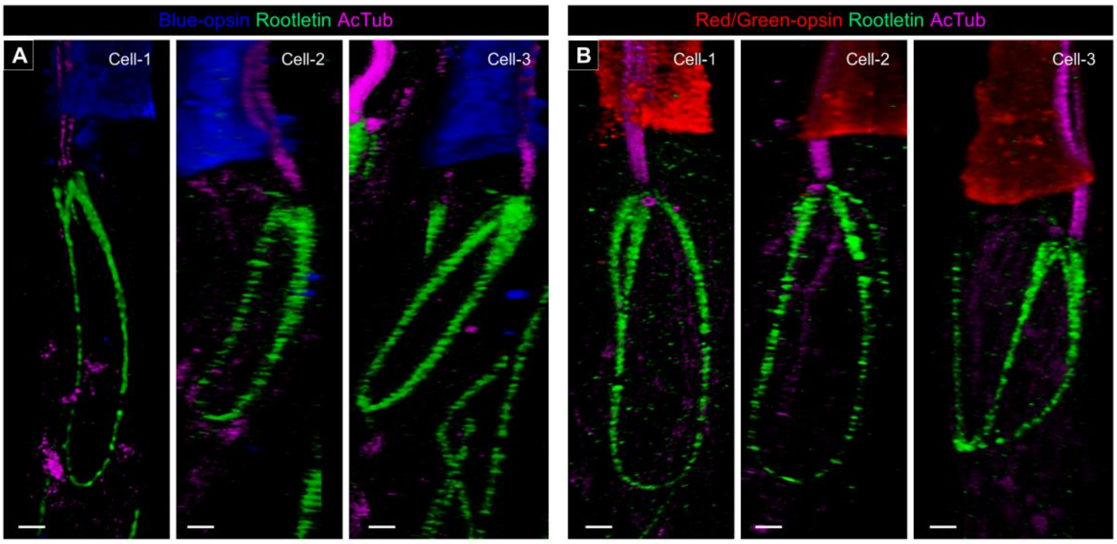

Supplementary Figure 6. Stricture of the PSC rootlet in normal canine blue and red/green cone photoreceptors

(A, B) 3D rendering images of U-ExM samples stained for rootletin (green) with blue-opsin (blue, A) or red/green-opsin (red, B). No significant differences are observed in the architecture of the PSC rootlet among the different cone subtypes. Scale bars: 500 nm, after correction for expansion factor. Abbreviation: PSC, photoreceptor sensory cilium.
